# Quantitative self-assembly of pure drug cocktails as injectable nanomedicines for synergistic drug delivery and cancer therapy

**DOI:** 10.7150/thno.55250

**Published:** 2021-03-31

**Authors:** Xiaona Chen, Binbin Xie, Lingling Huang, Jianqin Wan, Yuchen Wang, Xiaowei Shi, Yiting Qiao, Haihan Song, Hangxiang Wang

**Affiliations:** 1 The First Affiliated Hospital, Zhejiang University School of Medicine; NHC Key Laboratory of Combined Multi-Organ Transplantation; Key Laboratory of Organ Transplantation, Research Center for Diagnosis and Treatment of Hepatobiliary Diseases, Zhejiang Province, Hangzhou, PR China.; 2 Department of Medical Oncology, Sir Run Run Shaw Hospital, School of Medicine, Zhejiang University, Hangzhou, Zhejiang Province, 310003, PR China.; 3 Department of Chemical Engineering, Zhejiang University, Hangzhou, 310027, PR China.; 4 Central Laboratory, Shanghai Pudong New Area People's Hospital, Shanghai, 201299, PR China.

**Keywords:** self-assembly, drug cocktail, aggregation-induced emission, nanomedicine, synergistic combination.

## Abstract

New strategies to fabricate nanomedicines with high translational capacity are urgently desired. Herein, a new class of self-assembled drug cocktails that addresses the multiple challenges of manufacturing clinically useful cancer nanomedicines was reported.

**Methods:** With the aid of a molecular targeted agent, dasatinib (DAS), cytotoxic cabazitaxel (CTX) forms nanoassemblies (**CD NAs**) through one-pot process, with nearly quantitative entrapment efficiency and ultrahigh drug loading of up to 100%.

**Results:** Surprisingly, self-assembled **CD NAs** show aggregation-induced emission, enabling particle trafficking and drug release in living cells. In preclinical models of human cancer, including a patient-derived melanoma xenograft, **CD NAs** demonstrated striking therapeutic synergy to produce a durable recession in tumor growth. Impressively, **CD NAs** alleviated the toxicity of the parent CTX agent and showed negligible immunotoxicity in animals.

**Conclusions:** Overall, this approach does not require any carrier matrices, offering a scalable and cost-effective methodology to create a new generation of nanomedicines for the safe and efficient delivery of drug combinations.

## Introduction

Nanomedicines have great potential to provide effective treatment against devastating cancers by optimizing drug pharmacokinetics and tissue distributions, as well as reducing systemic drug exposure and associated toxicities 1. Over the past decades, versatile nanomaterials, including synthetic polymeric nanoparticles, have been developed for pharmaceutical delivery 2. To date, the success of these innovations has resulted in many nanosystems reaching clinical trials, and some of them have garnered FDA approval for human use 3, 4. Despite these applications, attempts to translate new nanomedicines into clinical use have to date encountered practical challenges. Therefore, pharmaceutical approaches with high translational capability are urgently desired. In our view, ideal nanosystems for cancer drug delivery should (1) have excellent drug loading capacity with minimum carrier matrices to reduce safety concerns; (2) achieve high encapsulation efficiency; (3) self-assemble into nanoparticles of appropriate size (usually < 200 nm in diameters); (4) enable the precise formulation of diverse drugs in single vehicles for combinatorial synergy; (5) be easily and reproducibly fabricated; (6) be scalable and cost effective; (7) avoid tedious schemes for drug synthesis and/or chemical modification; and (8) exhibit tolerable systemic toxicity and negligible immunotoxicity *in vivo* 5, 6. Unfortunately, most systems under development do not satisfy these criteria, and many intrinsic flaws remain (e.g., low drug loading capacity, heterogeneity of products, low batch-to-batch reproducibility, multistep synthetic and manufacturing protocols, safety concerns regarding biocompatibility, biodegradability and immunogenicity, and variations in the *in vivo* performance). In addition, the generation of nanomedicines often requires complex synthetic protocols involving both covalent conjugation and/or supramolecular nanoassembly, which significantly hinders the application of nanomedicines and may help explain the few successful examples in clinical practice. Hence, the design of a simple yet effective delivery system that easily moves into the clinic remains a challenging endeavor.

The molecular self-assembly of individual building blocks provides strategies for the creation of fascinating nanostructures for biomedical applications, especially in the field of drug delivery. In this context, “drug self-delivery systems” constructed from self-assembling small-molecule drugs should be promising nanomedicines 7, 8. However, most pure drugs are not applicable in this approach because of the inability to self-organize in aqueous media. Accordingly, on the basis of drug reconstruction, several sophisticated prodrugs have been designed to construct such self-assembling nanosystems. For example, the “squalenoylation” 9-11 or “PUFAylation” 12-14 of chemotherapeutic drugs provided the constructed prodrugs with the ability to self-assemble into nanoparticles in water, making them systemically injectable. In addition, several dimeric prodrugs composed of a hydrophobic drug and a hydrophilic drug ligated through cleavable linkages have been designed to create self-assembling amphiphiles 15, 16. While these strategies are effective, prodrug approaches may use tedious synthetic schemes. In some instances, a laborious trial-and-error process is required to optimize the linker chemistry or drug chemical modifiers to achieve favorable pharmacological activities 17. Therefore, self-deliverable systems assembled from single or multiple parent drugs could hold great potential for clinical translation due to the simplicity and feasibility in nanoparticle preparation 18, 19. Leveraging noncovalent interactions, some chemo drugs were capable of self-associating to form water-soluble nanoassemblies 20-22. For instance, amphiphilic doxorubicin (DOX) were reported to assemble with hydrophobic agents (e.g., 10-hydroxycamptothecin and celastrol) into hybrid nanoparticles driven by π-π stacking and hydrophobic interactions, enabling intravenous injection of drug combinations 23, 24.

Molecular targeted agents are widely successful as monotherapies or are rationally paired with standard chemotherapy for cancer treatment 25, 26. Combinatorial therapies have generally improved overall survival outcomes in the clinic. We noticed that some molecular inhibitors intrinsically exhibit amphiphilicity, which could allow this subset of inhibitors to self-assemble with hydrophobic drugs to form synergistic nanoassemblies for cancer therapy. To examine this hypothesis, we selected dasatinib (DAS) as a target compound to test whether DAS could assist the formation of nanoassemblies with cytotoxic taxane agents such as cabazitaxel (CTX).

**Figure 1:**
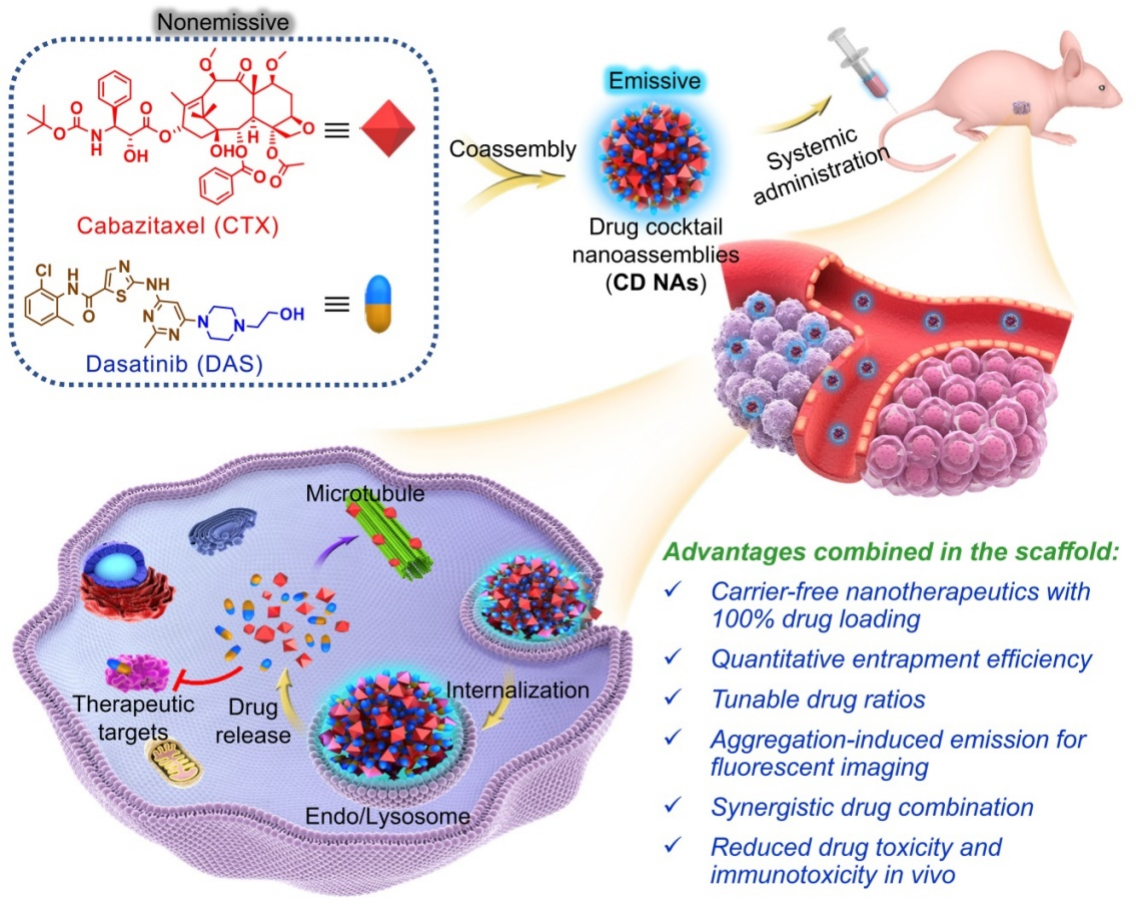
Schematic illustration of the fabrication of **CD NAs** from the coassembly of pure drug cocktails (cabazitaxel (CTX) and dasatinib (DAS)) for combination cancer therapy. The mixture of CTX/DAS in DMSO solvent is nonemissive, but upon aggregation, **CD NAs** emit intensive fluorescence, which can be used for intracellular nanoassembly trafficking. In addition, the **CD NAs** formulating dual drugs are suitable for intravenous injection. Once cellular uptake occurs, dual drugs with distinct modes of action can be released to exert their individual antitumor activities.

Exploiting the role of DAS as an amphiphile, we show here that DAS aids the self-assembly of water-insoluble, hydrophobic CTX into colloidally stable nanoassemblies without the need for exogenous excipients. Intriguingly, upon one-pot self-assembly process, nanoassemblies integrating pure drug cocktails (i.e., CTX and DAS) with tunable drug ratios are formed (termed **CD NAs**), offering the potential to codeliver bimodules for combination therapy (Figure 1). In this scaffold, we achieved nearly quantitative drug entrapment efficiency and 100% drug loading using a simple and practical protocol. Of note, we found that DAS molecules showed aggregation-induced emission (AIE) with a characteristic fluorescence emission at 422 nm, enabling the trafficking of **CD NAs** in cells without the additional labeling of fluorescent dyes 27. In two preclinical xenograft models, including a patient-derived xenograft (PDX) model, **CD NAs** exhibited significant synergy and enhanced efficacy to yield durable tumor recession. Further careful *in vivo* evaluation indicated that **CD NAs** alleviated systemic toxicity and evaded immunotoxicity, resulting from the advantages of small-molecule nanodelivery. Overall, this approach does not require any carrier matrices, providing a scalable and cost-effective methodology for the production of next-generation nanomedicines.

## Results and Discussion

### Self-assembly and characterization of CD NAs

**Figure 2:**
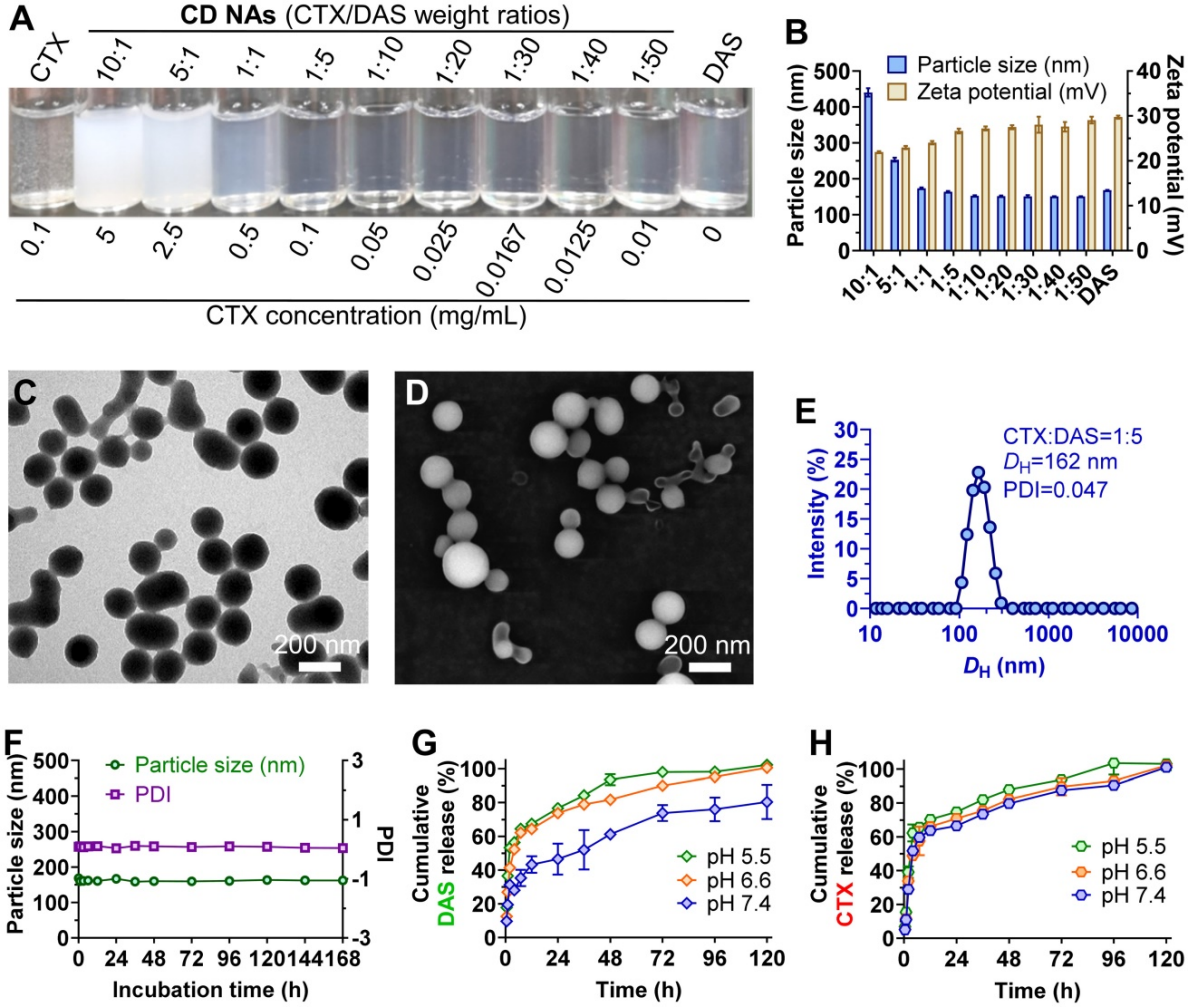
Characterization of self-assembled **CD NAs**. (A) Photograph of **CD NAs** fabricated at varying weight ratios of CTX to DAS. The nanoassemblies were prepared with a consistent DAS concentration at 0.5 mg/mL. The results indicate the formation of stable nanosuspensions. (B) A histogram of the particle size and zeta potential of **CD NAs** prepared at different weight ratios. (C) TEM and (D) SEM images of **CD NAs** (CTX/DAS, 1:5, w/w). Scale bars: 200 nm. (E) Size distribution of **CD NAs** determined by DLS analysis. The polydispersity index, PDI. (F) Variations in the particle size and PDI of **CD NAs** tested in PBS (pH 7.4, 10 mM). (G and H) *In vitro* release kinetics of DAS (G) and CTX (H) from **CD NAs** against PBS with different pH at 37 ℃. The data are presented as the means ± standard deviation (SD) of three independent experiments.

To assess whether DAS could coassemble with CTX, we quickly injected a solution of CTX/DAS in dimethyl sulfoxide (DMSO) into aqueous solution (e.g., deionized water). This procedure allowed the spontaneous formation of transparent solutions where both distinct mode-of-action drugs were coassembled, driven by multiple noncovalent interactions (Figure 1). Encouragingly, **CD NAs** could be fabricated at a broad range of weight ratios of CTX to DAS, from 10:1 to 1:50 (Figure 2A-B). In contrast, injection of the parent CTX in DMSO into water formed precipitates rather than transparent solutions. Because of the miscibility of the two agents, the drug ratios in the nanoassemblies could be precisely tuned in accordance with the optimized ratios from cell-based assays to achieve favorable combinatorial synergy. In contrast to the poor water solubility of CTX (~0.07 μg/mL) due to its bulky hydrophobic structure, we were able to prepare an aqueous suspension of CTX at concentrations up to 3 mg/mL when 50 wt% of DAS was coassembled (data not shown), representing at least a 42,857-fold increase in the solubility of CTX. Moreover, for self-assembled DAS, the solubility can be improved to 7.9 mg/mL, which is nearly 990 times higher than that of DAS in its free drug form (~8 μg/mL) 28. Currently, CTX is formulated with a blend of polysorbate 80 and ethanol under the trade name of Jevtana because of its water insolubility. Thus, this facile self-assembly protocol enables us to substantially improve drug solubility, rendering it injectable for preclinical investigation. Measurement of critical aggregation concentration (CAC) for **CD NAs** in aqueous media revealed that the CAC values were lower than 20 μg/mL (Figure S8), consistent with the CAC values of many surfactants 29.

Transmission electron microscopy (TEM) and scanning electron microscopy (SEM) were conducted to study the morphology of **CD NAs**. When **CD NAs** were assembled with a weight ratio of CTX to DAS at 1:5, the formation of uniformly spherical nanostructures was observed in both TEM and SEM images (Figure 2C-D). Such characteristic nanoassemblies with small sizes were also confirmed by SEM images when **CD NAs** were fabricated at other weight ratios (Figure S1). Dynamic light scattering (DLS) analysis further revealed that at varying ratios (CTX/DAS, w/w), sub-200 nm nanoassemblies with a narrow monomodal distribution were obtained (Table S1). The slightly larger sizes determined by DLS could be attributed to the hydration layer around the nanoassemblies and swelling in water, while the samples subjected to TEM or SEM measurements would encounter a shrinking process to form the anhydrous state. When we kept the concentration of DAS constant (0.5 mg/mL) for drug formulation, a decrease in CTX content made the particle solutions gradually become transparent (Figure 2A), and a reduction in particle sizes was observed (Figure 2B and Table S1). Moreover, to examine whether amphiphilic DAS molecule assisted the assembly of hydrophobic CTX, we gradually increased the DAS concentration while the CTX concentration was kept at 0.5 mg/mL. We found that 9 wt% of DAS was sufficient to solubilize CTX, forming a stable nanosuspension (Figure S2).** CD NAs** showed good colloidal stabilities in phosphate-buffered saline (PBS) and in PBS containing 10% (v/v) serum, without obvious changes in particle size and polydispersity index (PDI) for seven days (Figure 2F and Figure S3A). Moreover, the size of **CD NAs** did not vary when the DAS concentration was serially diluted from 1500 μg/mL to 50 μg/mL (Figure S3B). Furthermore, the particle sizes of **CD NAs** were temperature-independent at the range of 4℃ to 40℃ and even remained unchanged when incubated at 37℃ for 4 days (Figure S3C-F). Collectively, these data demonstrated the good stability of the nanoassemblies against physiologically relevant media as well as dilution and temperature.

Nanomedicines less than 200 nm in diameter potentiate passive targeting to tumors *via* the EPR effect, which enhances therapeutic efficacy and alleviates drug toxicity compared with that of solution-based free drug delivery 30. In addition, the particle surface was positively charged, as evidenced by the analysis of zeta potential, which may facilitate the intracellular uptake of nanoassemblies by cells 31. Moreover, the entrapment efficiency (EE) of CTX and DAS at varying weight ratios was determined. Impressively, the EE values for CTX and DAS were nearly quantitative (~99.9%, Table S1). The quantitative self-assembly of dual drugs provided a well-defined stoichiometry between CTX and DAS for *in vitro* and *in vivo* studies. Noticeably, using our pure drug self-assembling approach, an exceptionally high drug loading of up to 100% was achieved because no exogenous adjuvants were added.

Drug release profiles using **CD NAs** were further investigated against PBS at physiological pH (i.e., pH 7.4) or tumor acidic pH (i.e., pH 6.5 and 5.5). As shown in Figure 2G and 2H, **CD NAs** exhibited a pH-dependent release behavior of both drugs. Particularly, at pH 7.4, only ~40% of DAS and ~60% of CTX were released from **CD NAs** within an initial 12-h time period. However, the release of two drugs against pH 5.5 PBS was increased to ~70% in 12 h. The accelerated drug release could be explained by the fact that acidic solution attenuated the interactions between drug molecules, which could facilitate the drug diffusion across tumor sites and improve therapeutic efficacy.

### Mechanistic studies for molecular self-assembly

**Figure 3:**
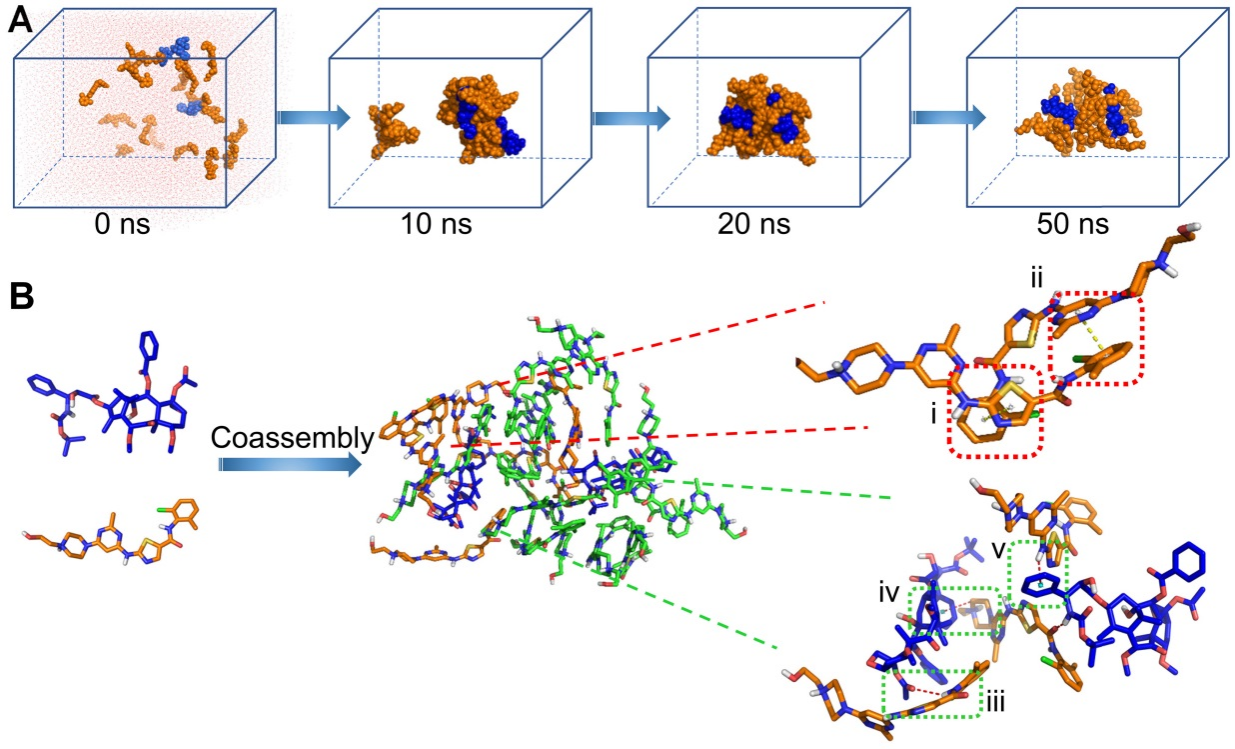
Molecular dynamics (MD) simulations of the CTX/DAS coassembled system. (A) Snapshots of the CTX/DAS aggregates show the self-assembly process after 10, 20, and 50 ns production simulations. (B) Typical non-covalent interactions between drugs: (i) and (ii) π-π stacking interactions endowed by the aromatic groups of the DAS molecules; (iii) amide N-H…O hydrogen bond; (iv) and (v) C-H … π and N-H … π interactions, respectively; and van der Waals interactions (not shown). CTX and DAS agents are colored in blue and orange, respectively.

To explore the self-assembly behavior of **CD NAs**, all-atom molecular dynamics (MD) simulations were carried out using the AMBER16 program 32. Mainly, the self-assembly process and the potential interactions between drug molecules were investigated. The molar ratio of CTX to DAS was fed at 2:17 (equals 1:5 at weight ratio), and initially, nineteen molecules were packed randomly in a cubic box with a length of 100 Å (Figure 3A). One snapshot of the aggregate was chosen from the simulation dynamics to depict the stacking manner of dual drug molecules in the aggregated state. In the initial state, all molecules dissociated randomly. The simulations resulted in the aggregation of drugs into a sphere-like compact architecture. Moreover, at a larger system size with increased number of molecules, MD simulations still resulted in the formation of the clusters, in which the hydrophilic motif of the DAS agent preferred to decorate the particle surface (Figure S4). During the simulation, we found that noncovalent interactions, including π-π stacking, hydrogen bonding, and van der Waals interactions, were driving forces for nanoassembly (Figure 3B). These interactions dominated the self-assembly of dual drugs and probably stabilized the overall nanosystem.

To further elucidate the interactions that contribute to drug coassembly, three agents were included to test the disruption of the nanoassemblies. Sodium chloride, urea, and surfactants (e.g., Tween 20, Triton X-100 and sodium dodecyl sulphate (SDS)) were known to attenuate electrostatic interaction, hydrogen bonding, and hydrophobic interaction, respectively 33, 34. The DLS results indicated that **CD NAs** were dissociated by Tween 20, Triton X-100 and SDS as a result of hydrophobic competition (Figure S5). Moreover, addition of urea into **CD NAs** also resulted in dramatic variations in the particle sizes and derived count rates. In contrast, sodium chloride was unable to impact the dissociation of the formed nanoassemblies. These experimental observations suggest that the driving force for drug coassembly was due to hydrophobic interaction and hydrogen bonding, in accordance with the results derived from MD simulations.

### AIE characteristics of CD NAs

**Figure 4:**
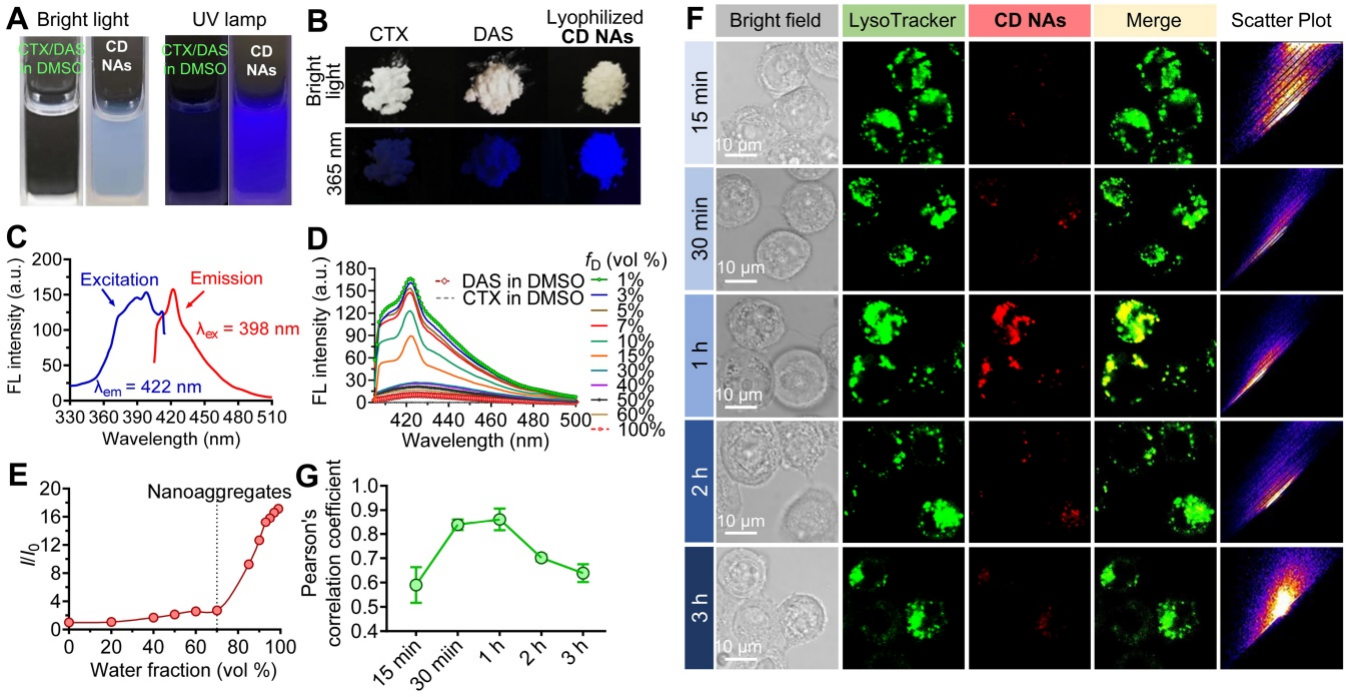
The AIE characteristics of **CD NAs**. Photographs of (A) the CTX/DAS mixture in DMSO and **CD NAs** in 10% (v/v) DMSO aqueous solution and (B) the original solid powder of CTX, DAS and the lyophilized **CD NAs**, taken under bright light or 365 nm UV irradiation with a UV lamp. (C) Emission and excitation spectra of **CD NAs** in water. (D) Emission spectra of the CTX/DAS mixture in DMSO/water solutions with different DMSO fractions (*f*_D_). (E) Plot of the relative FL intensity (*I*/*I*_0_) of **CD NAs** versus the water/DMSO mixture, where *I*_0_ denotes the FL intensity of the CTX/DAS mixture in DMSO solution. (F) Confocal images of cellular uptake and colocalization of **CD NAs** (red) in NCI-H1975 cells. Commercially available LysoTracker Green DND-26 was used to stain endo/lysosomes (green). Scale bars: 10 µm. (G) Pearson's correlation coefficient analysis of colocalization between **CD NAs** and endo/lysosome markers over time by ImageJ. The data are presented as the means ±SD, quantified with 4 independent fields of view.

The simple mixture of CTX and DAS dissolved in DMSO was nonemissive (Figure 4A and Figure S6), whereas the solutions containing **CD NAs** exhibited strong blue fluorescence under irradiation with a UV lamp. Strikingly, lyophilized **CD NAs** showed brighter emission under UV (365 nm) illumination ascribed to the preservation of intensely compact structures compared with the CTX or DAS powder (Figure 4B). This AIE was further validated by the fluorescence spectra (Figure 4C-D) 35. With a gradual decrease in the fraction of good solvent, i.e., of DMSO (*f*_D_), the fluorescence intensity of the mixture of CTX and DAS was dramatically increased under the excitation wavelength of 398 nm. For example, compared with the CTX/DAS mixture in pure DMSO solution (*I*_100_), the fluorescence intensity of CTX/DAS nanoaggregates in DMSO/water solutions with a 1% DMSO fraction (*I*_1_) was increased by ~17-fold (Figure 4E). Decreased DMSO fractions could promote the self-assembly of drug cocktails; thus, this phenomenon could be explained by the typical AIE effect endowed by the DAS molecules 36, 37. Detailed mechanistic studies showed that DAS served as an AIEgen and restriction of intramolecular motion contributed the AIE characteristics of the self-assembled DAS molecules 27.

To gain insight into the AIE behavior, UV absorption was examined. The UV spectra revealed that the coassembled **CD NAs** in water had a larger absorptivity derived from DAS (~326 nm) than the CTX/DAS mixture in DMSO (Figure S7). The increase in absorbance is associated with aggregate formation, probably indicating that these π-rich chromophore-like drugs in **CD NAs** are better conjugated than their individual counterparts in their free drug forms. We speculated that in DMSO, DAS undergoes intramolecular motion and dissipates exciton energy. Upon aggregate formation, the radiative pathway predominates strong fluorescence emission *via* the restriction of intramolecular motions 38, 39. While there are abundant AIE luminogens (AIEgens), this could be the first example to exploit molecular inhibitors (e.g., DAS) as an AIEgen 27, 40. Therefore, the AIE feature of the DAS agent could provide potential use for monitoring the intracellular behaviors of the overall nanoassemblies.

Intrigued by the AIE feature of **CD NAs**, we next examined whether this can be used to track the cellular uptake of nanoassemblies under confocal laser scanning microscopy (CLSM) 41. For this purpose, H1975 cells were exposed to **CD NAs**, and the resulting fluorescence images were observed using CLSM (Figure 4F). Only negligible fluorescence signals were detected after 15 min. After incubation for 1 h, the fluorescence signals derived from nanoassemblies were elevated inside the cells, indicating that **CD NAs** undergoes rapid cellular uptake and can be tracked by CLSM without the requirement of fluorescent dye labeling. Additionally, almost all of the nanoassemblies (red) appeared to colocalize with LysoTracker (green), a specific dye that labels endosomes/lysosomes, suggesting that **CD NAs** mainly accumulated in endo-lysosomal compartments (Figure 4F). After 2 h of incubation, the signals became weaker, presumably due to the disassembly of **CD NAs** in cells, which resulted in the release of free drugs accompanied by the partial loss of fluorescence. We further performed Pearson's correlation coefficient (PCC) analysis to quantitatively assess the colocalization of **CD NAs** with LysoTracker (Figure 4G). The analysis revealed a high PCC value of 0.86 ± 0.05 at the time point of 1 h, suggesting highly positive colocalization. A decline in PCC values was observed following further incubation. Together, these findings suggest that intrinsic AIE from DAS nanoaggregates can be used to monitor both nanoassembly trafficking and drug liberation in cells without additional labeling with fluorescent dyes.

### Comprehensive evaluation of synergism *in vitro*

**Figure 5:**
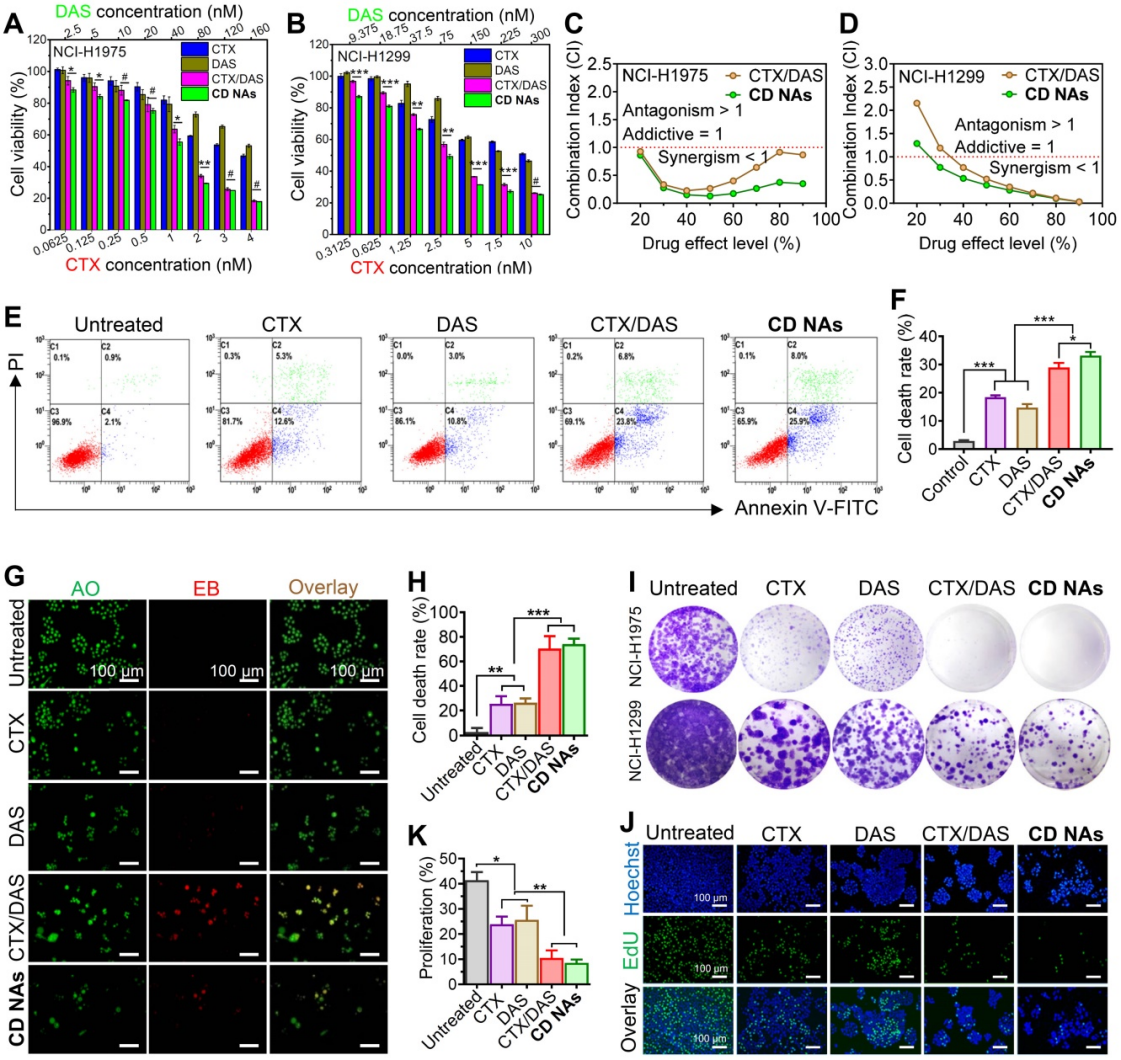
Synergistic combination of CTX and DAS in human non-small cell lung cancer NCI-H1975 and NCI-H1299 cell lines. (A and B) *In vitro* cytotoxicity of CTX, DAS, free CTX/DAS mixture, and** CD NAs** against both cancer cells as determined by the CCK-8 assay. (C and D) Comparative analysis of CI values using the CTX and DAS combination. Molar ratios of CTX to DAS were 1:40 and 1:30 in H1975 and H1299 cells, respectively. (E and F) Fluorescence-activated cell sorting (FACS) analysis of NCI-H1975 cell apoptosis induced by different treatments for 48 h. (G and H) Dual AO/EB staining assay for analyzing cell death in NCI-H1975 cells with quantification of apoptotic cells by counting the EB-positive cells. Scale bars: 100 µm. (I) Long-term colony formation assay. The cells were grown in the presence of drugs and photographed after staining with a 0.1% crystal violet solution. (J and K) Microscopic observation of the proliferation of NCI-H1975 cells treated with different drugs using a Click-iT EdU assay with quantitative results. Scale bar: 100 µm. The data are presented as the means ± SD of three independent experiments. *^#^p* > 0.05, *p < 0.05, **p < 0.01, ***p < 0.001.

To assess the combinatorial activity, we first carried out Cell Counting Kit-8 (CCK8) assays to examine cell viability and investigate the synergistic effects against human non-small cell lung cancer NCI-H1975 and NCI-H1299 cell lines. In this experimental setting, free CTX, free DAS, and the combination of individual drugs CTX/DAS (i.e., CTX and DAS were added to cell culture separately) were included as references (Figure 5A-B). As expected, CTX exhibited potent cytotoxicity, with half-maximal inhibitory concentrations (IC_50_) of 4.6 nM in H1975 cells and 13.1 nM in H1299 cells, whereas the IC_50_ values of the DAS agent were 194.8 nM and 392.3 nM in H1975 and H1299 cells, respectively (Figure S9 and Table S2). Thus, we fixed the molar ratios of CTX to DAS at 1:40 and 1:30 in H1975 and H1299 cells, respectively, for further investigation. In both cell lines, a substantially reduced IC_50_ of CTX was achieved after combining CTX with DAS. For example, the IC_50_ of CTX decreased from 4.6 to 0.7 nM in H1975 cells and from 13.1 to 2.6 nM in H1299 cells when tested with the **CD NA** platform, indicating that DAS increased the sensitivity of cells to CTX. In addition, compared with the mixture of CTX/DAS, **CD NAs** were demonstrated to be more potent in both tested cell lines. These data suggest the synergy of using combinatorial **CD NAs** against cancer cells.

To further quantitatively confirm the synergism of CTX/DAS, we conducted a combination index (CI) determination (see experimental details in Supplementary Material). The synergistic effects were examined among varying molar ratios of CTX to DAS from 1:40 to 1:1 in NCI-H1975 cells. The CI values are extrapolated from the dose-response curves of the drug combination, where CI values less than, equal to, or greater than 1 represent synergism, additivity, or antagonism, respectively 42. As presented in Table S3, drug combination at varying ratios resulted in satisfactory synergism, with CI values less than 0.6. Notably, compared with the simple CTX/DAS mixture, co-assembled **CD NAs** had lower CI values, indicating an excellent combinatorial synergism (Table S3). In particular, either free CTX/DAS drug mixture (CI = 0.393) or **CD NAs** (CI = 0.231) showed the most potent synergy when the drug ratio of CTX/DAS was fixed at 1:40. Accordingly, **CD NAs** fabricated at this ratio was used for next assays in NCI-H1975 cells.

We further plotted CI values against drug effect levels, which can offer quantitative information about the extent of drug interactions. As shown in Figure 5C-D, all of the CI values of the drug combination were lower than 1 after 48 h of incubation of the drugs in H1975, supporting the synergism of the CTX/DAS combination. Noticeably, **CD NAs** showed CI values less than 0.5 over a wide range of drug effect levels (IC_90_ through IC_30_), exhibiting stronger synergy than the free CTX/DAS drug mixture.

To gain insight into whether cell death was a consequence of apoptosis induced by **CD NAs**, we performed FITC-Annexin V/propidium iodide (PI) double staining. H1975 cells were incubated with various drugs for 48 h and then subjected to flow cytometry analysis. The results showed that the ratios of apoptotic cells were 18.37%, 14.73%, and 28.93% after treatment with CTX, DAS, or the CTX/DAS mixture, and the apoptotic ratio increased to 33.17% when the cells were incubated with **CD NAs** (Figure 5E and F). Similarly, in AO/EB assays, **CD NAs** promoted a much higher apoptotic rate of H1975 cells than the other treatments (Figure 5G and H). Previous studies demonstrated that targeting Src with its small-molecule inhibitors (e.g., DAS) could increase the efficacy of standard chemotherapeutics in breast cancer cells 43. Therefore, we further deciphered the synergistic mechanism by which the combination of Src inhibition and CTX treatment increases apoptotic cell death. Western blot analysis showed that upon DAS exposure, the phosphorylation levels of Src as well as its downstream signal transducers and activators of transcription 3 (STAT3) were decreased (Figure S10) 44. Specifically, in the cells treated with the CTX/DAS mixture or **CD NAs**, p-STAT3 expression was substantially abrogated, indicating the efficient inhibition of oncogenic pathways activated by p-Src. We further assessed the level of apoptotic factors. Treatment with **CD NAs** resulted in the upregulation of apoptotic c-PARP, Bax and p53, whereas antiapoptotic Bcl-2 and survivin were attenuated in the cells (Figure S11).

The colony formation assay was further conducted to evaluate the long-term activity of **CD NAs**. Each colony is derived from a single cell, and therefore, the result reflects the ability of chemotherapies to prevent the cells from dividing. After drug exposure, the cells were stained with crystal violet and then photographed (Figure 5I). Significantly fewer colonies survived on the plates exposed to **CD NAs** than in the plates treated with free CTX or free DAS. To further verify whether short-term cell proliferation can be inhibited by **CD NAs**, we utilized EdU incorporation to label the cells undergoing DNA synthesis. Treatment with **CD NAs** for 48 h resulted in greatly impaired proliferation in H1975 cells (Figure 5J and K), whereas monotherapy treatment produced only negligible antiproliferation activity. Thus, the combination was more effective than the free individual drugs in the inhibition of cell proliferation. Together, these data provide compelling evidence that dual drugs (i.e., DAS and CTX) can be rationally combined in a single scaffold to produce therapeutic synergy. Coassembled **CD NAs** were more effective than the combination of free drugs, which was probably attributable to efficient cellular internalization of the assemblies.

### Examination of near-infrared (NIR) fluorescence imaging of CD NAs

The biodistribution of **CD NAs** was investigated in NCI-H1975 tumor bearing Balb/c mice to evaluate the tumor-targeting capacity of the nanoassemblies. Here, a hydrophobic NIR dye Cy5.5 was used to label **CD NAs** *via* a co-assembly protocol. When the tumor volume reached 500 mm^3^, the mice were intravenously injected with Cy5.5-labeled **CD NAs** (Cy5.5@**CD NAs**), and free Cy5.5 was injected as control at an identical dosage. The biodistribution of **CD NAs** was examined using fluorescence imaging at predetermined time points. As shown in Figure S12, NIR signals ascribed to Cy5.5@**CD NAs** accumulated in the tumor regions during the observation study for 48 h. However, in the free Cy5.5-treated mice, whole-body NIR signals decayed rapidly, and negligible fluorescence was observed in the tumors. The mice were sacrificed at 48 h post-injection, and the tissues were subjected to *ex vivo* imaging (Figure S13). Of note, the intensity of NIR fluorescence in the tumors of the mice receiving **CD NAs** was 3.1-fold higher than that of free Cy5.5, providing the evidence that the CD NA platform enabled the preferential tumor accumulation than the free drug form (Figure S13C).

### Antitumor efficacy of CD NAs in two separate preclinical mouse models

**Figure 6:**
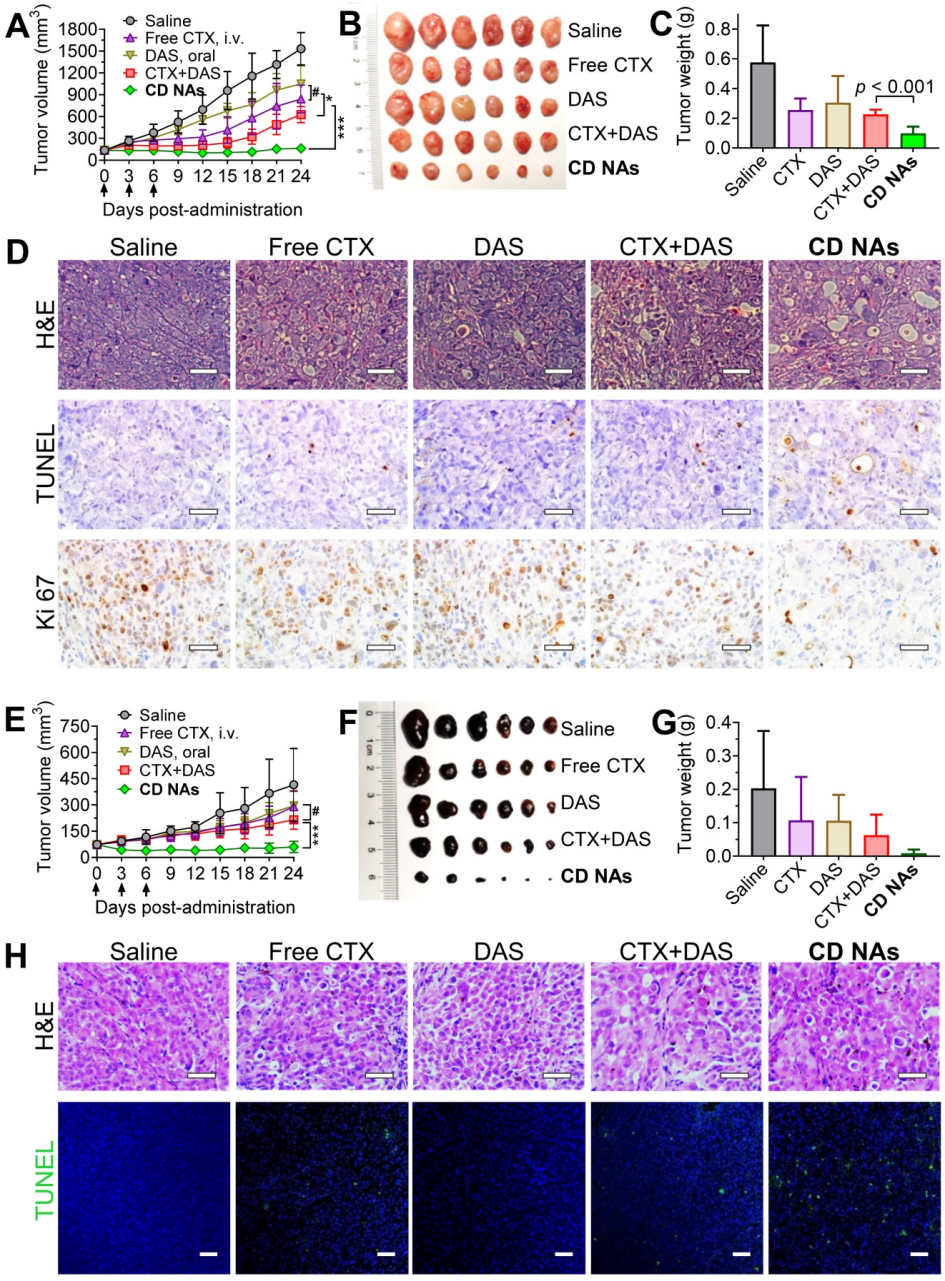
The synergistic efficacy of **CD NAs** in mouse models bearing human lung H1975 tumors (A-D) and melanoma PDX tumors (E-H). Tumor growth curves (A and E), photographs of the tumors excised from the mice (B and F), and the tumor weights at the endpoint of the therapeutic studies (C and G). Each mouse was intravenously administered drugs *via* the tail vein as indicated by the arrows. (D) Representative images for H&E staining, TUNEL assay and Ki67 immunohistochemistry in H1975 tumor sections after the treatments. Scale bars: 50 µm. (H) H&E staining and TUNEL analysis of the excised tumors from different groups in the mouse model bearing melanoma PDX at the end of the therapeutic studies. Scale bars: 50 µm. The data are presented as the means ± SD (n = 6). *p < 0.05, **p < 0.01, ***p < 0.001, ^#^*p* > 0.05.

Encouraged by the combinatorial synergy from *in vitro* results and the potentially improved bioavailability of these nanomedicines *via* intravenous (IV) administration, we examined the therapeutic efficacy of **CD NAs** in preclinical mouse models. An NCI-H1975 cancer cell-derived xenograft (CDX)-bearing mouse model was established in immunodeficient Balb/c nude mice by subcutaneously implanting cancer cells into the right flank of the mice. When the tumors reached 50-100 mm^3^ in volume, the mice were randomly divided into five groups (n = 6) and received various treatments: (1) saline (IV injection); (2) free CTX (as a pharmaceutical Jevtana-mimicking formulation, polysorbate 80 and ethanol, 3 mg/kg, IV); (3) free DAS (15 mg/kg, *via* gavage); (4) combination therapy of individual free CTX and DAS [CTX+DAS, coadministered as free CTX (3 mg/kg, IV) and free DAS (15 mg/kg, *via* gavage)]; (5) **CD NAs** (IV, 3 mg/kg CTX and 15 mg/kg DAS). The routes of free CTX and DAS were both guided by clinical usage, and the mice were given three injections every three days. As shown in Figure 6A, the tumor volumes in the saline group increased rapidly, reaching an 11.4-fold increase at the experimental endpoint. The free drugs alone had a modest impact on tumor progression. The **CD NAs** outperformed the free CTX and free DAS treatments in terms of tumor control, producing durable tumor suppression. Noticeably, the repression of tumor growth in **CD NA**-treated mice was substantially more effective than that of the mice receiving the combination regime of free drugs. For example, the tumor growth inhibition (TGI) rate in the mice treated with **CD NAs** was 89.5%, which was statistically better than that of the other treatments (i.e., the inhibition rates for CTX, DAS and the free drug combination group were 45.2%, 31.5%, and 59.1%, respectively). A TGI exceeding 40% is regarded as an effective treatment. Therefore, the observations were consistent with the *in vitro* experiments, indicating that the tumors exhibited a noticeable response to the nanomedicines. The tumor weights obtained from each group on day 24 are shown in Figure 6C, which is consistent with the tumor growth curve, and a typical photograph of the tumors excised from the mice is shown in Figure 6B. The survival study showed that the mice received **CD NAs** had prolonged survival compared with that of the free drug combination (Figure S14).

Given the promising efficacy in the CDX model, we further evaluated the potential of **CD NAs** in a PDX mouse model, which was established as previously described 45. Briefly, the metastasized melanoma specimen was surgically resected from a patient with melanoma. We then finely sectioned the tumor tissue into approximately 1 mm^3^ pieces and implanted it subcutaneously into the right flanks of immune-compromised Balb/c nude mice to produce orthotopic melanoma PDX. As depicted in Figure 6E-G, in combination with orally administered DAS, IV injection of free CTX in the Jevtana-mimicking formulation impeded tumor growth more effectively than CTX or DAS treatment alone, but the differences were not statistically significant. Previous reports showed that the oral bioavailability of DAS ranged from 14% in mice to 34% in dogs 46. Thus, the minimal synergism could be explained by the low bioavailability of DAS due to oral administration, which resulted in insufficient plasma concentrations mostly outside the narrow therapeutic window in this melanoma PDX model 47, 48. Encouragingly, **CD NAs** resulted in remarkable tumor shrinkage relative to the CTX+DAS combination therapy (Figure 6E). After the injection of **CD NAs**, the tumor volumes were reduced by half relative to that of day 0, which indicates strong synergy. Noticeably, the therapeutic durability lasted throughout the 18-day treatment-free period. Furthermore, measurement of the tumor weight at the endpoint revealed that the tumor inhibition rate was increased to 95.67% when treatment with **CD NAs** was applied. Accumulating evidence has shown that PDX models have powerful predictive ability to assess drug response and activity *in vivo*. Upon passaging in mice, the derived tumor xenografts are expected to retain the key characteristics of the donor tumors with regard to genetics and histology. While most preclinical drug evaluations have relied on *in vitro* assays and *in vivo* CDX transplantation models, we validated the efficacy of **CD NAs** in a human melanoma PDX model. In contrast to CDX models, PDXs are established from individual patients and thus could recapitulate morphological and genetic features of the donor tumors. Therefore, we anticipate that this clinically relevant PDX may mirror the clinical responses of human melanoma to treatment with **CD NAs**.

Furthermore, a TUNEL assay of the tumors excised from each group revealed that extensive histological features of intratumoral apoptosis were induced by **CD NAs**. This result correlated well with the hematoxylin and eosin (H&E) staining (Figure 6D and 6H, the panels of H&E and TUNEL). In addition, immunohistochemical Ki-67 staining was used to assess the proliferation of tumor cells after various treatments. The smaller number of positive cells in **CD NA**-treated mouse tumors implied the superior inhibition of proliferation (Figure 6D, the panels of Ki67). In summary, we can safely conclude that **CD NAs** show remarkable antitumor efficacies in both CDX and PDX models.

### Evaluation of the safety profiles of CD NAs *in vivo*

**Figure 7:**
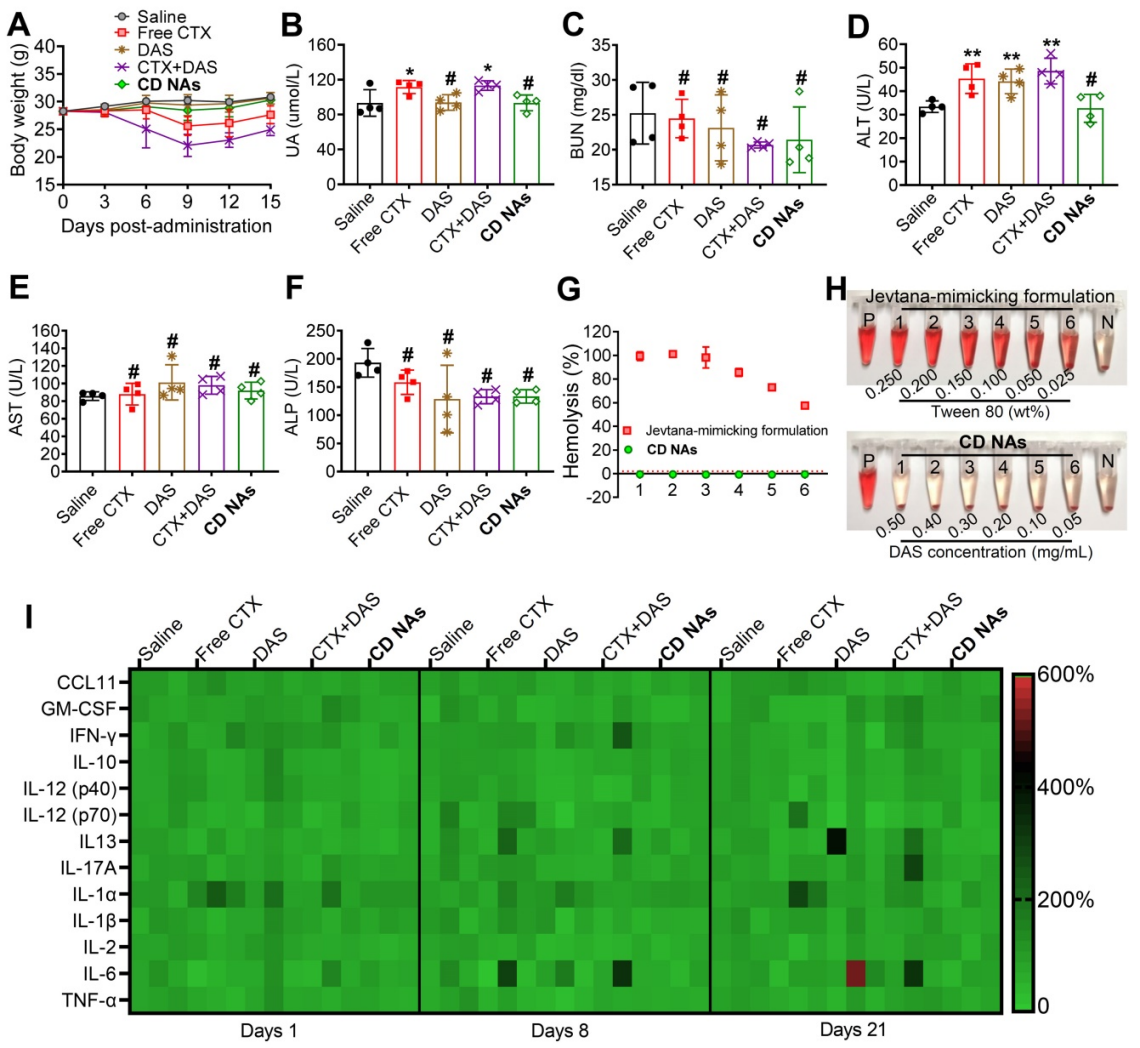
Systemic toxicity and immunotoxicity in healthy ICR mice after systemic administration of **CD NAs**. (A) Changes in body weights measured every 3 days. (B-F) Serum biochemistry profile of mice to evaluate drug-induced dysfunction of kidney (UA and BUN) and liver (ALT, AST and ALP) after three injections. UA, uric acid; BUN, blood urea nitrogen; ALT, alanine aminotransferase; AST, aspartate aminotransferase; ALP, alkaline phosphatase. (G and H) Relative hemolysis rates of RBC suspensions upon incubation with **CD NAs** or free CTX (as a pharmaceutical Jevtana-mimicking formulation) at the same CTX concentrations. Tubes P and N denote the positive control (Triton X-100, set as 100%) and the negative control (PBS, set as 0%), respectively. The concentrations of CTX from 1 to 6 were 0.10, 0.08, 0.06, 0.04, 0.02, and 0.01 mg/mL. (I) Heat map of the relative expression of mouse cytokines following treatment. The data are presented as the means ±SD (n = 3-4); *p < 0.05, **p < 0.01, ***p < 0.001, and ^#^*p* > 0.05 versus the saline group.

Toxicity is a primary consideration for the further clinical translation of nanomedicines. We thus carefully evaluated a series of toxicological pathologies to provide an overall safety profile for **CD NAs**. First, we monitored the variation in body weights in each treatment group. Clinical CTX formulation was reported to show high toxicity in phase I clinical trials. As expected, the administration of free CTX (6 mg/kg) resulted in a substantial reduction in average body weight, with weight loss of ~15.3% and ~26.9% in the CTX- and CTX/DAS-treated mice, respectively, on day 9. This result revealed that free CTX elicited significant toxicity in animals (Figure 7A). Very impressively, there was no observed difference in body weights between the mice treated with **CD NAs** and the mice treated with saline throughout the study, indicating that the toxicity could be alleviated by our injectable nanomedicines.

Hematological studies were also utilized to evaluate the potential toxicity of **CD NAs** after systemic administration. Specifically, leukopenia and neutropenia are dose-limiting toxicities caused by free CTX in its clinical formulation, which is characterized by reduced white blood cell (WBC), lymphocyte (LY), and neutrophil (NE) counts 49. As shown in Table S4, the majority of the key hematological constituents, such as red blood cells (RBCs), in the mice receiving various formulations remained within the normal range. Unfortunately, the administration of either CTX or the combination of CTX/DAS exhibited significant neutropenia/leukopenia, as evidenced by the reduced WBC, LY and NE counts in the mice. Impressively, **CD NAs** induced a negligible reduction in the aforementioned parameters, and they all rebounded to the normal range after cessation of treatment (Table S4). Furthermore, serology was analyzed after three injections to evaluate acute toxicity to the liver and kidney (Figure 7B-F). The absence of signs of toxicity was validated in the **CD NA**-treated mice, whereas abnormal elevations in uric acid (UA) and alanine aminotransferase (ALT) were detected in the mice exposed to treatments such as free CTX or free drug combination. As a critical renal indicator, increasing UA implies potential renal toxicity. Moreover, the elevated levels of ALT in the mice could be explained by hepatic dysfunction after treatment with CTX, DAS, or the free drug combination.

The improved drug tolerance of using **CD NAs** was further supported by histological analysis. The mice treated with the free drug combination presented the signs of necrosis or cell death in the liver and lung, indicating the potential damage to these organs (Figure S15). To our delight, no histopathological damage of the major organs was found in the mice that received saline or** CD NAs**. A possible explanation for the reduced toxicity could be that drug molecules delivered by **CD NAs** are constrained within a reservoir during blood circulation, thereby sparing healthy organs and tissues from toxic free drug exposure.

To increase the solubility of hydrophobic agents, surfactants are generally used as pharmaceutical excipients. Polysorbate 80, a typical surfactant, is an essential excipient to formulate CTX but may induce hemolysis of RBCs. Thus, we carried out a hemolysis assay to examine the hemolytic effect of **CD NAs** in comparison with CTX in the Jevtana-mimicking formulation. As a result, a hemolysis rate of more than 50% was observed upon treatment with the Jevtana-mimicking formulation, which was ascribed to the presence of only 0.025% (v/v) polysorbate 80, and complete lysis of the erythrocytes was observed in the presence of a proportion higher than 0.150% (v/v) (Figure 7G-H). Encouragingly, a hemolysis rate of less than 1% was observed after the administration of **CD NAs**, indicating no detectable disturbance of the red blood cell membranes. Taken together, these studies suggest the low toxicity of self-assembled **CD NA** scaffolds, indicating their great potential for clinical translation.

The immunotoxicity of drug-loaded nanosystems remains a major roadblock that impedes their further clinical applications. Previous studies indicate that activation of the immune system is strongly associated with the hydrophobicity of nanoparticles 50, 51. Traditional (co)polyester-based nanoparticles generally possess high hydrophobicity and fail to evade immunotoxicity, which could also partially cause the difficulty of clinical translation 52. We hypothesize that the present novel nanomedicines constructed from self-assembling small-molecule drugs should hold the potential to avoid immune response during systemic administration. To validate our hypothesis, a panel of inflammatory and pro-inflammatory cytokines (e.g., CCL-11, GM-CSF, IFN-γ, IL-10, IL-12 (p40), IL-12 (p70), IL-17A, IL-1α, IL-1β, and TNF-α) and T helper 1/2-related cytokines (e.g., IL-2, IL-6, IL-13 and TNF-α) in mouse serum were measured after IV injection of various drug formulations at a clinically relevant dosage. Thereafter, serum samples from the mice were collected, and cytokine levels were analyzed using a multiplex cytokine assay to evaluate acute or long-term immunotoxicity. Compared with the healthy mice, the serum levels of the cytokines in the **CD NA**-treated mice were in the normal range after 24 h, suggesting that **CD NAs** were able to circumvent acute immunotoxicity (Figure 7I). Moreover, no apparent difference in the cytokine levels was observed on day 8 or 21 after three successive injections. These results implied the long-term safety of **CD NAs** for the immune system.

## Conclusion

In conclusion, we have presented a proof-of-principle demonstration of a new generation of nanomedicines constructed from a self-assembling pure drug cocktail composed of the cytotoxic drug CTX and the molecular inhibitor DAS. **CD NAs** can be fabricated *via* a simple self-assembly process with quantitative entrapment efficiency, 100% drug loading, tunable ratios of the drug cocktail, markedly enhanced drug solubility, potent antitumor efficiency, and improved safety profiles *in vivo*. Furthermore, the AIE property ascribed from DAS molecules could be exploited for particle trafficking and drug release analysis in living cells. We anticipate that with further screening of molecular inhibitors, other drug combinations could be accessible using these pure drugs self-assembly approach. Overall, our approach might address the design and scalability challenges posed by the combinatorial delivery of multiple drugs with distinct modes of action. Given the intrinsic advantage of CTX in circumventing drug resistance and its therapeutic synergy in combination with DAS, we aim to initiate early-phase clinical trials to assess the safety and efficacy of this robust nanomedicine in patients with taxane-resistant cancer.

## Materials and Methods

### Preparation and characterization of CD NAs

Briefly, the self-assembled **CD NAs** were prepared using the antisolvent method. To prepare the **CD NAs** at various weight ratios of CTX to DAS, 10 μL of CTX solution at predetermined concentrations in DMSO and 10 μL of DAS solution (10 g/L) in DMSO were blended, and then the mixture was smoothly injected into 180 µL of DI water under ultrasonication. Prior to further use, **CD NAs** solution was subjected to centrifugation (2,000 g, 5 min) to remove precipitates and was dialyzed against DI water to remove free drugs and organic solvent DMSO. The drug concentrations were then determined by analytic reverse-phase high-performance liquid chromatography (RP-HPLC). The hydrodynamic diameters (*D*_H_), size distribution and zeta potential were determined by dynamic light scattering (Malvern Nano-ZS90, Malvern, UK). TEM and SEM studies were performed to observe the morphology of the **CD NAs**. A small drop of the sample solution (1 mg/mL DAS coassembled with 0.2 mg/mL CTX) was spread on a 300-mesh carbon-coated copper grid. After 10 min, filter papers were used to remove the primary solvent. After drying at room temperature for 15 min, NAs were characterized under a transmission electron microscope (TECNAL 10, Philips) or scanning electron microscope (Nova Nano 450, Thermo FEI). The absorbance of the free CTX, free DAS, and free CTX/DAS mixture in DMSO and of **CD NAs** was determined by a UV/vis spectrophotometer (UV-2700, Shimadzu). A fluorescence spectrometer (Cary Eclipse, Agilent Technologies) was used to measure the fluorescence intensity of the coassembled NAs in water in a 1.0 cm quartz cuvette.

### Determination of EE

The entrapment efficiency of the nanomedicines was determined by analytical HPLC. Briefly, **CD NAs** were centrifuged to collect the supernatants and precipitates, and the contents of the dual agents were subsequently quantitatively determined by HPLC. HPLC was performed using a Hitachi Chromaster 5000 system with a YMC-Pack C8 column (5 μm, 250 × 4.6 mm) at a flow rate of 1.0 mL/min. All of the runs used linear gradients of acetonitrile (solvent A) and water (solvent B) containing 0.1% trifluoroacetic acid (TFA). The EE of CTX and DAS in coassembled NAs was calculated according to the following equation:

EE (%) = W_drug in NAs_/W_feed_ × 100%, where W_drug in NAs_ and W_feed_ denote the amount of CTX or DAS encapsulated in NAs and the corresponding CTX or DAS amount fed for NA fabrication, respectively.

### *In vitro* drug release

To investigate the release kinetics of DAS and CTX from **CD NAs**, 3 mL of **CD NAs** fabricated with DAS (0.5 mg/mL) and CTX (0.1 mg/mL) was dialyzed against 20 mL of PBS supplemented with 0.3% Tween 80 at different pH. The dialysis tubes (Spectrum, molecular weight cutoff of 7 kD) were stirred continuously in an orbital shaker at 37 ℃. At predetermined time intervals, the release media were collected and fresh media were supplemented. The amounts of released DAS and CTX were measured by HPLC.

### Cell lines and cell culture

Two human non-small cell lung cancer cell lines, NCI-H1975 and NCI-H1299, were acquired from the Cell Bank of the Chinese Academy of Sciences. At 37 °C with 5% CO_2_, the NCI-H1975 cells were maintained in DMEM, while NCI-H1299 cells were incubated in RPMI 1640 medium, both supplemented with 10% fetal calf serum (FBS) and 1% penicillin plus streptomycin. The experiments were performed until the confluence of cells reached 75%.

### Establishment of the NCI-H1975 CDX mouse model

Mice were injected subcutaneously in the right flank region with 100 µL of a cell suspension containing 1 × 10^6^ NCI-H1975 cells. When the tumor size reached approximately 100 mm^3^, the NCI-H1975 tumor-bearing mice were randomly divided into five groups, and the mice in different groups (n = 6) were treated with (1) saline (intravenous injection [IV]); (2) free CTX (formulated in polysorbate 80/ethanol (1:1, v/v), 3 mg/kg, IV); (3) free DAS (formulated in DMSO/water (1:1, v/v), 15 mg/kg, *via* gavage); (4) individual free CTX and DAS [CTX+DAS, coadministered as free CTX (3 mg/kg, IV) and free DAS (15 mg/kg, *via* gavage)]; or (5) self-assembled **CD NAs** (3 mg/kg CTX and 15 mg/kg, IV), administered every 3 days for a total of 3 doses. Each mouse was marked and followed individually throughout the whole experiment. The tumor size (length and width) of the mouse was measured before every injection. Tumor volume (V) was calculated as follows: V (mm^3^) = 1/2 × length (mm) × width (mm)^2^. Mice were sacrificed at the end of the experiment, and the tumors of the mice were excised, photographed and weighed.

### Establishment of a human melanoma PDX model

The melanoma PDX model used in this study was established at the Key Laboratory of Combined Multi-Organ Transplantation, Ministry of Public Health, School of Medicine, Zhejiang University, as previously described 45. With patient's informed consent (No.1730670), the resected human melanoma tissue was obtained surgically and then collected in an ice bath in RPMI 1640 supplemented with antibiotics. We initiated drug treatment using the fourth generation of this melanoma PDX model. The tumor tissue was diced into ~1 mm^3^ pieces and subcutaneously implanted into the right flanks of the nude mice within 2 h as the engraftment phase. For serial transplantation, the mice bearing PDX tumors were sacrificed, and ~1 mm^3^ tumor fragments were further implanted into ~4-week-old nude mice. Drug treatment was performed starting in the third generation. When the PDX tumor volumes reached 50-100 mm^3^, the mice (n = 6 in each group) were administered different treatments by a regimen identical to that used in the NCI-H1975-xenograft model with three successive doses at days 0, 3, and 6. Saline was intravenously injected as a control. The volumes of the tumors were monitored. The mice were sacrificed by CO_2_ inhalation at the end point.

### System toxicity study

ICR mice were randomly divided into five groups (n = 4-5) and received various treatments: (1) saline (IV); (2) free CTX (6 mg/kg, IV); (3) free DAS (30 mg/kg, *via* gavage); (4) individual free CTX and DAS [CTX+DAS, coadministered with free CTX (6 mg/kg, IV) and free DAS (30 mg/kg, *via* gavage)]; or (5) **CD NAs** (IV, 6 mg/kg CTX and 30 mg/kg DAS) three times every three days. All mice under different treatments were humanely sacrificed at the end point, and whole blood or serum were harvested for subsequent testing. For hematological studies, blood was collected in a tube containing heparin and counted using a blood counter system (Celltac ES, NIHON KOHDEN). For serology analysis, urea nitrogen, alanine aminotransferase, aspartate aminotransferase and alkaline phosphatase were measured to indicate renal and liver functions at day 8 after the first administration. Independently, body weights were monitored and recorded every three days starting from the treatment for 15 days.

### Statistical analysis

Data are presented as the mean ± SD unless otherwise indicated (*p < 0.05, **p < 0.01, ***p < 0.001, ^#^*p* > 0.05). One-way ANOVA and a two-tailed Student's *t* test were performed in SPSS 17.0 software. Data were compared with the saline control group and between groups unless otherwise indicated.

## Supplementary Material

Supplementary figures and tables.
