# Supplementary material for: Quantitative self-assembly of pure drug cocktails as injectable nanomedicines for synergistic drug delivery and cancer therapy
Source: Theranostics. 2021 Mar 31;11(12):5713–27. doi: 10.7150/thno.55250 (PMC8058735; doi:10.7150/thno.55250)
Supplement: Supplementary file 1 — Supplementary figures and tables. [file thnov11p5713s1.pdf]

Supplementary Material

Quantitative self-assembly of pure drug cocktails as injectable nanomedicines for synergistic drug delivery and cancer therapy

Xiaona Chen[1], Binbin Xie[2], Lingling Huang[1], Jianqin Wan[1], Yuchen Wang[1,3], Xiaowei Shi[1], Yiting Qiao[1], Haihan Song[4,], and Hangxiang Wang[1,*]*

[1] The First Affiliated Hospital, Zhejiang University School of Medicine; NHC Key Laboratory of Combined Multi-Organ Transplantation; Key Laboratory of Organ Transplantation, Research Center for Diagnosis and Treatment of Hepatobiliary Diseases, Zhejiang Province, Hangzhou, PR China.

[2] Department of Medical Oncology, Sir Run Run Shaw Hospital, School of Medicine, Zhejiang University, Hangzhou, Zhejiang Province, 310003, PR China.

[3] Department of Chemical Engineering, Zhejiang University, Hangzhou, 310027, PR China.

[4] Central Laboratory, Shanghai Pudong New Area People's Hospital, Shanghai, 201299, PR China.

** Corresponding authors: Hangxiang Wang, The First Affiliated Hospital, Zhejiang University School of Medicine; 79, Qingchun Road, Hangzhou 310003, China. E-mail: wanghx@zju.edu.cn*

Haihan Song, Central Laboratory, Shanghai Pudong New Area People's Hospital, Shanghai, 201299, PR China. E-mail: haihansong@163.com

Materials

Cabazitaxel (CTX) was purchased from Knowshine Pharmachemicals Inc. (China). Dasatinib (DAS) and Cell Counting Kit-8 were obtained from MedChemExpress (MCE; Shanghai, China). Near-infrared fluorescent probe Cy5.5-azide was purchased from Lumiprobe Corporation (Hallandale Beach, FL, USA). The Click-iT® EdU Alexa Fluor® 488 Assay Kit was purchased from Invitrogen (Shanghai, China). The AO/EB Dual Fluorescence Staining Kit was purchased from Solarbio (Beijing, China). The Annexin V-FITC/PI Apoptosis Detection Kit was obtained from Dojindo Laboratories (Shanghai, China). The Src rabbit mAb (catalog no: 2109), phospho-Src family (Tyr416) rabbit mAb (catalog no: 2101), STAT3 rabbit mAb (catalog no: 4904), phospho-STAT3 rabbit mAb (catalog no: 94994), Bax rabbit mAb (catalog no: 5023), p53 rabbit mAb (catalog no: 2527), Bcl-2 mouse mAb (catalog no: 15071), survivin mouse mAb (catalog no: 2802), c-PARP rabbit mAb (catalog no: 5625), GAPDH rabbit mAb (catalog no: 2118) and β-actin rabbit mAb (catalog no: 8457) were purchased from Cell Signaling Technology, Inc. (Danvers, MA, USA). Deionized (DI) Milli-Q water (Millipore) was used throughout the experiments.

Solubility test

To determine the solubility for DAS, 50 mg of DAS was incorporated into the nanoparticles in 5 mL of DI water. The resultant suspension was stirred for 30 min at 25 ° C. After centrifugation for 3 min at 5000 rpm, the amount of DAS in the supernatant was measured by UV/vis spectroscopy at a wavelength of 326 nm.

Determintation of critical aggregation concentration (CAC)

CAC was determined using the fluorescence intensity change upon aggregates formation in aqueous solution [1]. Before the aggregates formation, the fluorophores exhibit very weak fluorescence. Once the aggregates were formed, the fluorescence intensity increased significantly. This method was used to determine the CAC of **CD NAs** and fluorescence intensity at 422 nm was plotted against the DAS concentrations in different media such as DI water, PBS and FBS (10%, v/v) at room temperature.

Molecular dynamics simulations

The structures of CTX and DAS were optimized, and the partial atomic charges were calculated by the restrained electrostatic potential charge from the calculation with the Gaussian09 package at the level of HF/6-31g* [2]. MMFF94x force field parameters were used for these two molecules. CTX and DAS molecules were mixed at a molar ratio of 1:8.5 (CTX: DAS) and initially packed randomly by PACKMOL in a cubic box with a length of 100 Å [3]. Then, the mixture was neutralized by adding sodium/chlorine counterions and solvated in a cuboid box of TIP3P water molecules with 10 Å solvent layers between the box edges and solute surface.

All MD simulations were performed using AMBER16 [4]. The AMBER GAFF force field was applied, and the SHAKE algorithm was used to restrict all covalent bonds involving hydrogen atoms with a time step of 2 fs. The Particle mesh Ewald method was employed to treat long-range electrostatic interactions. For each solvated system, two minimization steps were performed before the heating step. The first 4000 cycles of minimization were performed with all heavy atoms restrained with 50 kcal/(mol·$Å^2$), whereas solvent molecules and hydrogen atoms were able to move freely.

Then, nonrestrained minimization was carried out with 2,000 cycles of steepest descent minimization and 2,000 cycles of conjugated gradient minimization. Afterwards, the whole system was first heated from 0 K to 300 K in 50 ps using Langevin dynamics at a constant volume and then equilibrated for 400 ps at a constant pressure of 1 atm. A weak constraint of 10 kcal/(mol·$Å^2$) was used to restrain all the heavy atoms during the heating steps. Periodic boundary dynamics simulations were carried out for the whole system at NPT (pressure = 1 atm, and temperature = 300 K) in the production step. In the production phase, a 50 ns simulation was carried out.

Evaluation of cell viability by CCK-8 assay

NCI-H1975 and NCI-H1299 cells were seeded ($2\sim3 \times 10^3$ cells per well) into a 96-well plate and cultured overnight until the cells were fully attached. Various concentrations of CTX, DAS, CTX+DAS, and **CD NAs** in medium were added to each well. After coincubation with different drugs for 48 h, the medium was replaced with fresh 10% (v/v) CCK-8 solution, and the cells were further incubated for 2 h at 37 ° C. The plates were then analyzed using a microplate reader (Multiskan FC, Thermo Scientific) with the absorbance set at 450 nm. Untreated cells in medium were used as a control. Each experiment was performed at least three times.

Synergistic effect evaluated by the combination index (CI)

The degree of synergy between two drugs can be quantified by calculating the CI using Calcusyn 2.1 (Biosoft, Cambridge, U.K.). The CI values of CTX combined with DAS were calculated from the dose-effect profiles according to the equation: $CI = D_1/Dm_1 + D_2/Dm_2$, where D_1 and D_2 represent the concentrations of drug 1 and drug 2, respectively, that in combination produce a certain level of cytotoxicity, and Dm_1 and Dm_2 are

the concentrations of the single drugs, administered separately, that produce the same effect. CI values less than 1 indicate synergism, CI values equal to 1 indicate an additive effect, and CI values larger than 1 indicate antagonism.

Cellular uptake of CD NAs

We utilized CLSM to detect the cellular uptake of self-assembled **CD NAs** in NCI-H1975 cells. The cells were plated in 20 mm glass coverslips at 2.0 $\times 10^5$ cells per well and incubated for 24 h. Then, the solution containing **CD NAs** (40 μg/mL DAS coencapsulated with 1 μg/mL CTX) was added. The cells were processed after 15 min, 30 min, 1 h, 2 h and 3 h of incubation with two washes in PBS and then stained with LysoTracker Green (100 $\times 10^{-9}$ M) for 30 min at 37 ° C. After washing, the cells were immediately observed using a confocal microscope (FV3000, Olympus, Japan). The images were further analyzed with ImageJ (NIH, USA).

Apoptosis assay

Cell apoptosis assays were performed by Annexin V-fluorescein isothiocyanate (FITC)/propidium iodide (PI) double staining in NCI-H1975 cells. Briefly, cells were seeded in 6-well plates (3.0 $\times 10^5$ cells per well) and incubated for 24 h until fully attached. Then, cells were treated with either CTX (2 nM), DAS (80 nM), CTX+DAS (2 nM CTX plus 80 nM DAS) or **CD NAs** at the same concentrations for 48 h. Cells were stained with an Annexin V-FITC Assay Kit (Dojindo Laboratories, Japan) and then measured by flow cytometry (Cytomic FC 500MCL, BECKMAN COULTER). For each sample, 1.0 $\times 10^4$ events were collected.

EdU assay to detect cell proliferation

NCI-H1975 cells were seeded in flat-bottomed 48-well plates (1.5 ×

10^4 cells per well) and incubated at 37 ° C for 24 h. Free CTX (1 nM), free DAS (40 nM), CTX+DAS (1 nM CTX/40 nM DAS) or **CD NAs** at the same concentrations were added to the cells and then incubated for 24 h at 37 ° C. The Click-iT® EdU Alexa Fluor® 488 Assay Kit was used to quantify the cell proliferation rate according to the manufacturer's protocol. At the end of the drug exposure, EdU (5-ethynyl-2'-deoxyuridine) was added to each well, and the cells were further incubated for 2 h at 37 ° C. The cells were washed with PBS and fixed for 15 min at room temperature by the addition of 4% formaldehyde. Upon incubation with 0.5% Triton X-100 for 10 min, Alexa Fluor® 488 was added and incubated for another 30 min in the dark. The nuclei were stained with Hoechst 33342 (provided in the kit) for 15 min, and the cells were imaged by fluorescence microscopy (IX73, Olympus). Finally, three regions in each group were counted to assess cell proliferation.

Acridine orange/ethidium bromide (AO/EB) staining to detect cell death

NCI-H1975 cells were cultured in flat-bottomed 48-well plates (1.5 × 10^4 cells per well) at 37 ° C for 24 h and then coincubated with either free CTX (1 nM), free DAS (40 nM), the CTX+DAS mixture (1 nM/40 nM) or **CD NAs** at the corresponding concentrations. After treatment, 1 µL of dye mix (1 µg/mL acridine orange plus 1 µg/mL ethidium bromide in PBS) was added to the cells. Observation was carried out at 200× using a fluorescence microscope (IX73, Olympus). The cells in three regions were counted, and the number of cells with fragmented nuclei, increased cytoplasm and condensed chromatin reliably indicated apoptosis.

Colony formation assay

A colony formation assay was conducted to evaluate the long-term

cytotoxicity of various drugs. NCI-H1975 or NCI-H1299 cells at a density of 1000 cells per well were seeded in flat-bottomed 12-well plates and incubated at 37 ° C. After 24 h of incubation, the cell medium was replaced by 5% FBS medium containing either CTX (0.6 nM), DAS (24 nM for H1975 cells or 18 nM for H1299 cells), the mixture of CTX/DAS, or **CD NAs** at the same concentration of single agents. After exposure to the drugs for 48 h, the cells were washed with PBS and replaced with fresh 10% FBS medium. After another 8 days, the cells were fixed, stained with 0.1% crystal violet and photographed.

Western blotting

NCI-H1975 cells were cultured in 6-well plates at a seeding density of 2.0×10^5 cells per well for 24 h. Next, the cells were incubated with medium containing free CTX (1 nM), free DAS (40 nM), CTX+DAS (1 nM/40 nM), or **CD NAs** (1 nM/40 nM). After 24 h of treatment, the cells were washed with precooled PBS, and then RIPA lysis buffer (Biyuntian, China) and complete protease inhibitor cocktail (Thermo Scientific, USA) were added to lyse the cells on ice for 30 min. The cell lysate was centrifuged at 15,000 rpm for 5 min at 4 ° C, and the supernatant was transferred to a new microtube for further detection. The total protein amount of the cell extracts was measured using a Pierce BCA Protein Assay Kit (Thermo Scientific, USA). SDS-polyacrylamide gel electrophoresis was carried out with 20-30 μg of protein lysate/lane from the sample, and then the gels were transferred to PVDF membranes (Millipore, USA). After blocking and incubation with primary and secondary antibodies, specific proteins were detected using an ECL Western Blotting Detection Kit (GE Healthcare Bioscience, UK). Images were taken using a gel imaging system (5200, Tanon, China) and analyzed with ImageJ software (NIH, USA).

Animal study

Male Balb/c athymic nude mice (4-5 weeks) were purchased from Shanghai Experimental Animal Center, Chinese Academy of Science, and housed at the animal facility of the Zhejiang Academy of Medical Sciences. The studies involving animals were approved by the Ethics Committee of the First Affiliated Hospital, Zhejiang University School of Medicine. All animal experiments were performed in compliance with the guidelines of the Zhejiang University Committee for Animal Use and Care.

In vivo and *ex vivo* imaging study

To evaluate the tumor-targeting ability and *in vivo* drug distribution of **CD NAs**, NCI-H1975 mouse model was established by subcutaneous injection of 1×10^6 NCI-H1975 cells into the right flank of Balb/c nude mice (4-5 weeks old). When the tumor volume reached ~500 mm^3, the mice were randomly divided into two groups (n = 5 in each group). A near-infrared dye Cy5.5 was coassembled into **CD NAs** (termed Cy5.5@**CD NAs,** DAS: CTX: Cy5.5 = 5:1:1, weight ratio) to track the *in vivo* distribution of **CD NAs**. The mice were injected with free Cy5.5 or Cy5.5@**CD NAs** at a Cy5.5 dose of 20 μg per mouse *via* the tail vein. Whole-body NIR fluorescence imaging was performed using an *in vivo* imaging system (IVIS® Lumina LT Series III, PerkinElmer, USA) at predetermined time points. At 48 h, the mice were sacrificed to collect tumors and organs for *ex vivo* imaging.

In vitro hemolysis

The blood compatibility of the self-assembled nanomedicines was investigated according to a previous report [5]. Blood was collected in heparinized tubes from healthy SPF SD rats, followed by centrifugation at 1500 rpm for 10 min. The pellet was washed three times with cold PBS (pH

7.4) by centrifugation at 1500 rpm for 10 min and resuspended in the same buffer. **CD NAs** or free CTX (as a pharmaceutical Jevtana-mimicking formulation, polysorbate 80 and ethanol) at the same CTX concentrations, also prepared in PBS buffer, were added to the erythrocytes and incubated for 60 min at 37 ° C in a shaking water bath. The procedure of free CTX as a pharmaceutical Jevtana-mimicking formulation was as follows: 40 mg of CTX was dissolved in polysorbate 80 and then diluted with 13% ethanol to give the final concentration. Untreated normal physiological saline and 0.2% Triton X-100 served as negative (yielding 0% values) and positive (yielding 100% values) controls. Finally, the optical density (OD) of the supernatant fluid was obtained by a microplate reader (Multiskan FC, Thermo Scientific) at 570 nm. The hemolysis ratio (HR) was calculated using the following equation: HR (%) $=[(OD_t - OD_n)/(OD_p - OD_n)] \times 100\%$. The OD_t indicates the OD value of the tested group. The OD_n and OD_p were the OD values of the negative and positive groups, respectively. Three samples were used in each group. Less than 2% hemolysis was regarded as a nontoxic effect level in our experiments.

Immunohistochemical analysis

The tumor-bearing mice were killed at day 12 post-administration. The tumors were excised, fixed in 10% paraformaldehyde and embedded in paraffin. The tissues from NCI-H1975 tumor xenograft-bearing mice were then sectioned for histopathological analysis by H&E staining, Ki67 and TUNEL immunohistochemistry, while the tissues from melanoma PDX tumors were stained with H&E and TUNEL immunofluorescence. The sections were imaged using an Olympus Microscope (IX73, Olympus) or confocal microscope (FV3000, Olympus, Japan).

Immunotoxicity assay

ICR mice were divided into five groups (n = 3) and treated with the same administration regimen used in the system toxicity study. At days 0, 1, 8, and 21 after administration, blood samples were allowed to clot for 30 min and then centrifuged at 14,000 rpm for 10 min. Then, the supernatant sera were collected, frozen and stored at -80 ° C. Before testing, the samples were further thawed and centrifuged at 13,000 rpm for 10 min at 4 ° C. Twenty-five microliters of serum was diluted with assay buffer (25 μL), which was provided in the LEGENDplex™ Multi-Analyte Flow Assay Kit (BioLegend Inc.). Serial dilutions of chemokines and cytokine standards were prepared in the Matrix (provided in the kit). Control serum, standards, and serum samples of mice treated with various drugs were incubated with antibody-immobilized beads for 30 min and then subjected to flow cytometry (Cytoflex S, Beckman Coulter, USA) according to the manufacturer's instructions.

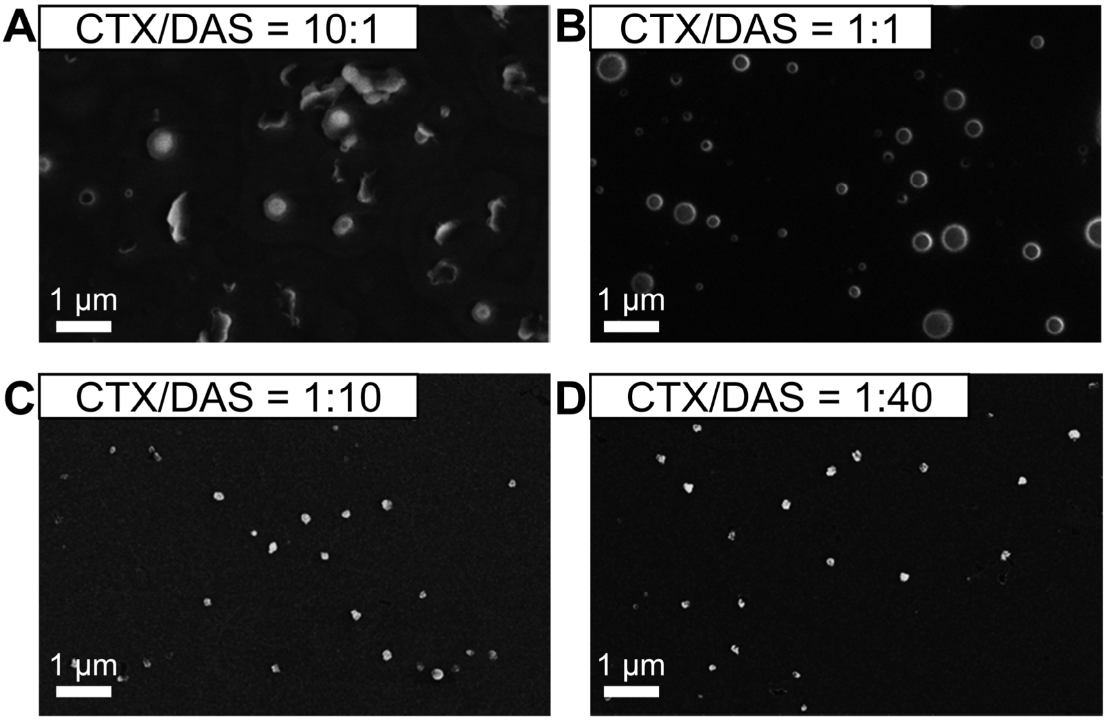

Figure S1. Scanning electron microscopy (SEM) images of **CD NAs** assembled from different weight ratios of CTX to DAS (10:1, 1:1, 1:10, and 1:40). When we increased the feed ratio of amphiphilic DAS agent, the particle sizes of **CD NAs** became smaller. Scale bars, 1 μm.

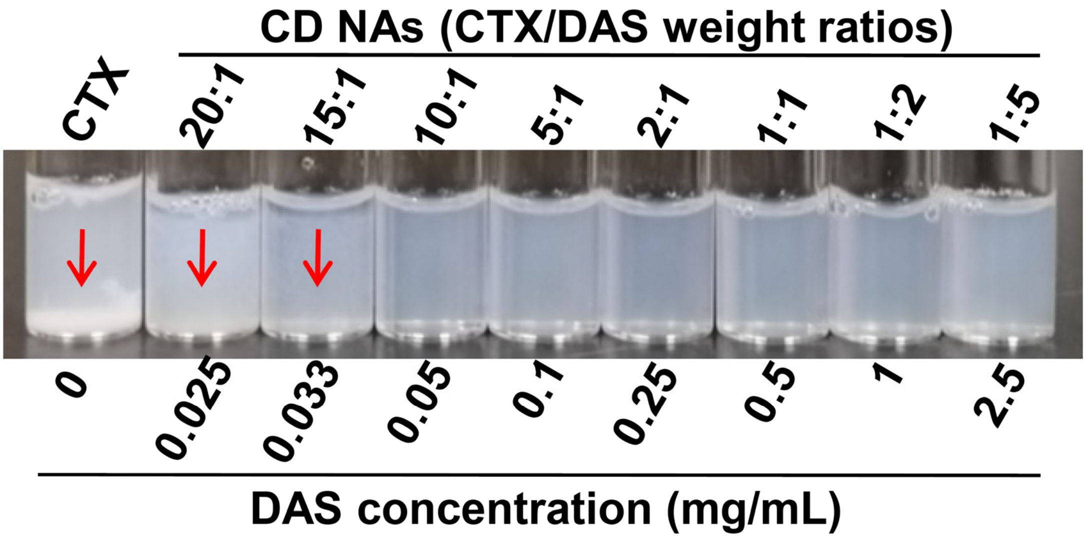

Figure S2. Photograph of **CD NAs** fabricated at varying weight ratios of CTX to DAS. The nanoassemblies were prepared with a consistent CTX concentration at 0.5 mg/mL. Hydrophobic CTX was solubilized to form stable nanosuspensions upon addition of DAS. The red arrows indicate the opaque precipitates.

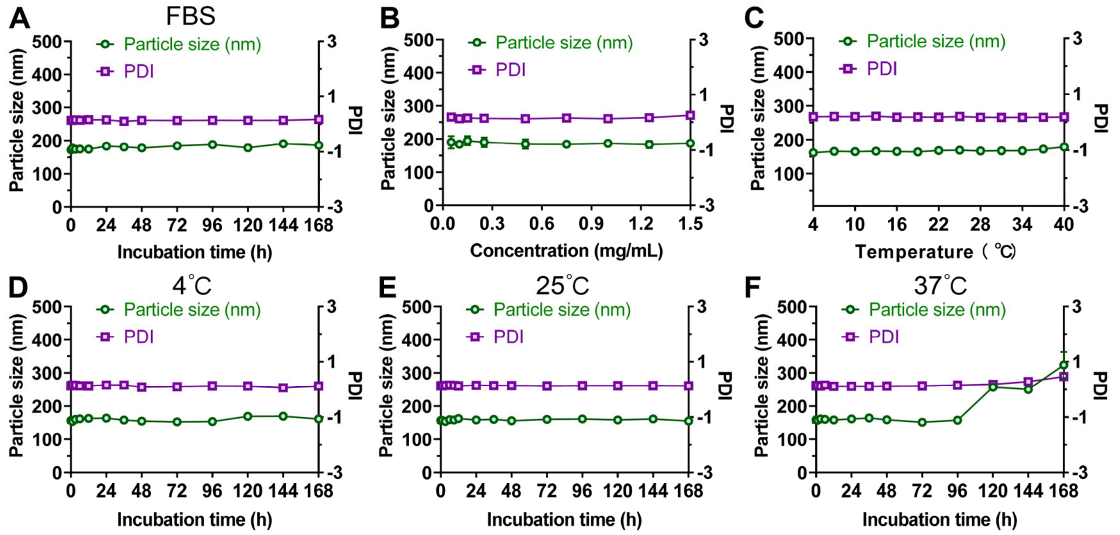

Figure S3. (A) Colloidal stability of **CD NAs** in the medium containing 10% fetal bovine serum (FBS). (B) Hydrodynamic diameters of **CD NAs** following the gradient dilution. (C) Temperature-independent stability of **CD NAs**. (D-F) Changes of hydrodynamic diameters of **CD NAs** when incubated at 4, 25, and 37 °C in DI water. The hydrodynamic sizes and zeta potentials were measured by DLS over the time course of 7 days. The data are presented as the means ± standard deviation (SD) (n = 3).

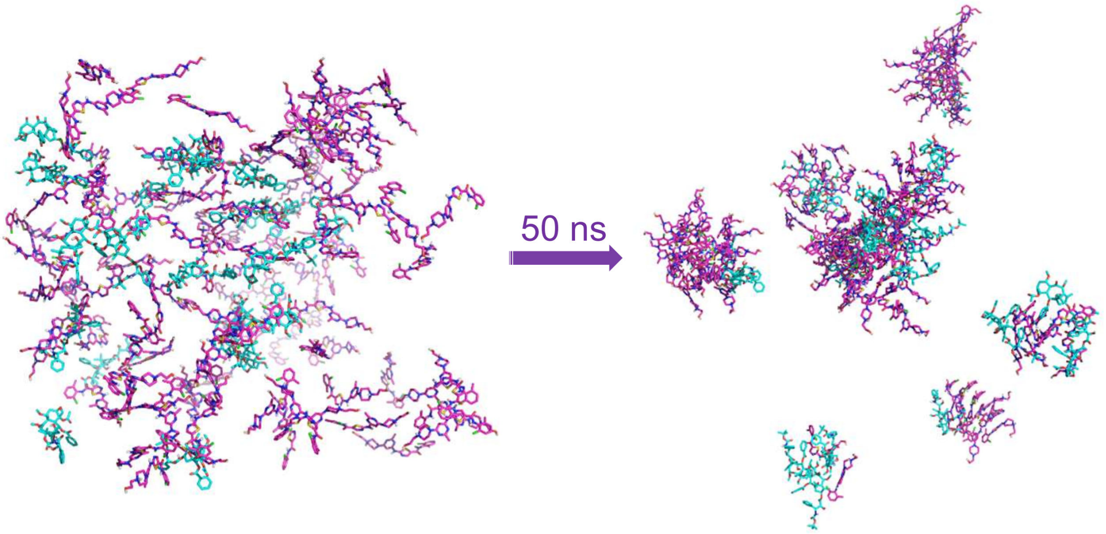

Figure S4. Snapshots of the CTX/DAS aggregates show the self-assembly process after 50 ns of molecular dynamics (MD) simulations. CTX and DAS are colored in cyan and purple, respectively.

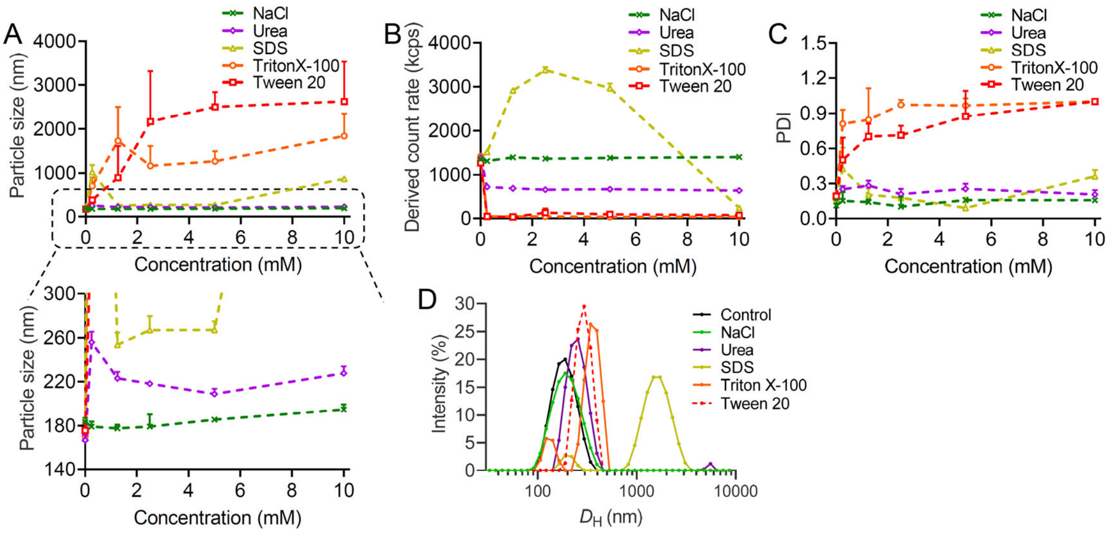

Figure S5. Dissociation of **CD NAs** induced by various agents. Sodium chloride, urea, and surfactants (e.g., Tween 20, Triton X-100 and sodium dodecyl sulphate (SDS)) were included for the investigation. Variations in particle size (A), derived count rate (B), and PDI (C) of the nanoassemblies after the treatment with various agents. The analyses were performed on Malvern Nano-ZS90 (Malvern, UK). The data are presented as the means ± SD (n = 3). D) Size distribution of **CD NAs** (CTX: 0.05 mM; DAS: 0.25 mM) measured by DLS in the presence of different agents.

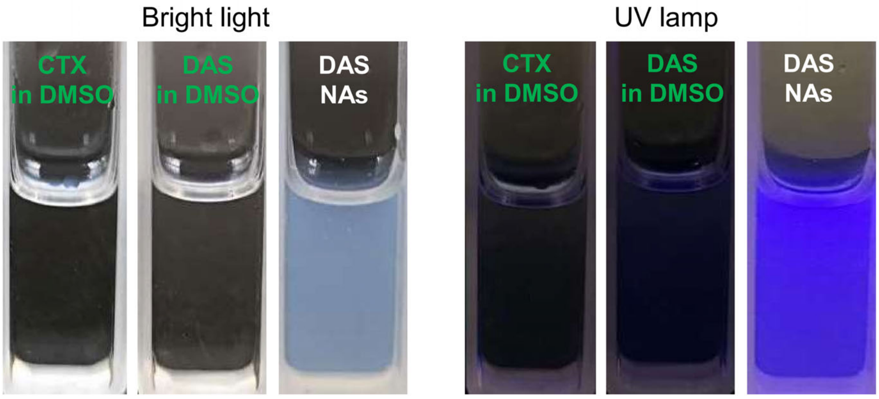

Figure S6. Photographs of free CTX, free DAS in DMSO and DAS NAs in 10% (v/v) DMSO aqueous solution. CTX concentration: 0.2 mg/mL; DAS concentration: 1 mg/mL.

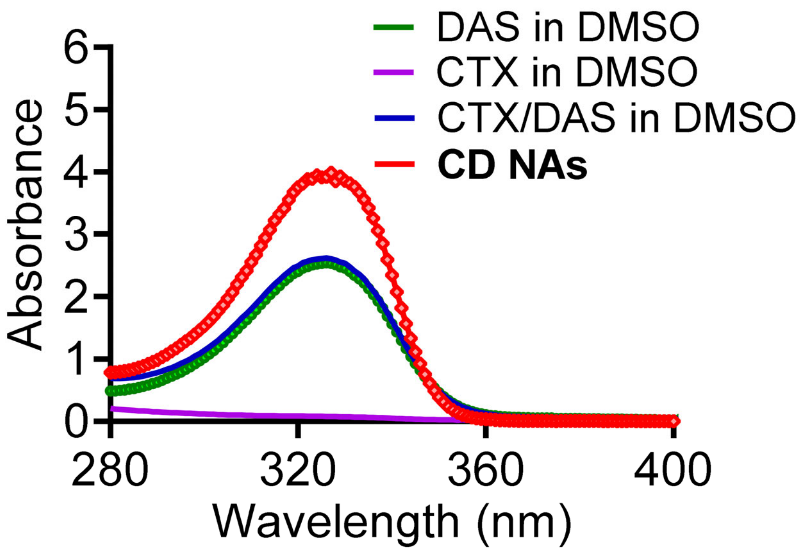

Figure S7. UV-vis absorption spectra of free CTX, free DAS, CTX/DAS mixture in DMSO, and **CD NAs** in 10% DMSO aqueous solution. In all samples, the CTX concentration was 0.2 mg/mL, while that of DAS was 1 mg/mL.

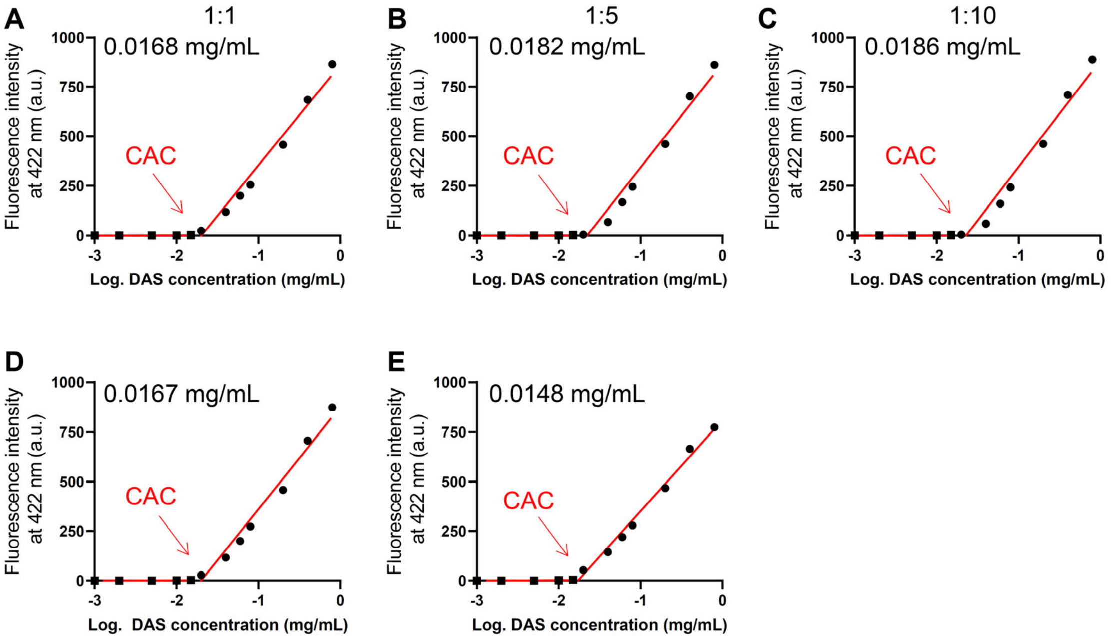

Figure S8. (A-C) Critical aggregation concentration (CAC) of **CD NAs** at different weight ratios of CTX to DAS in DI water. (D-E) CAC determination of **CD NAs** (CTX/DAS=1:5, w/w) in PBS (pH 7.4, 10 mM) (D) and in PBS containing 10% (v/v) serum (E). Fluorescence intensity at 422 nm (excited at 398 nm) was used for data plotting.

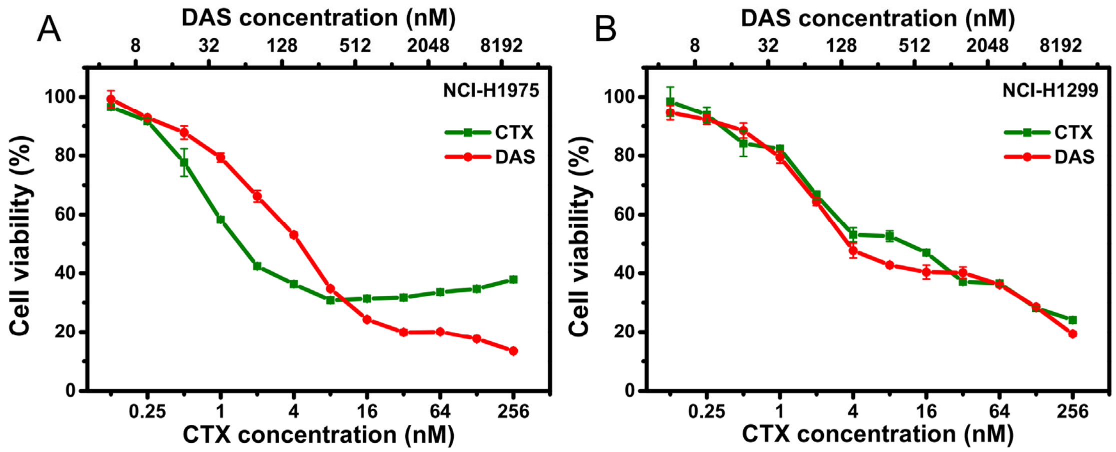

Figure S9. *In vitro* cytotoxicity of CTX and DAS against human non-small cell lung cancer cell lines NCI-H1975 (A) and NCI-H1299 (B) as determined by the CCK-8 assay. The data are presented as the means ± SD, quantified with 3 independent experiments.

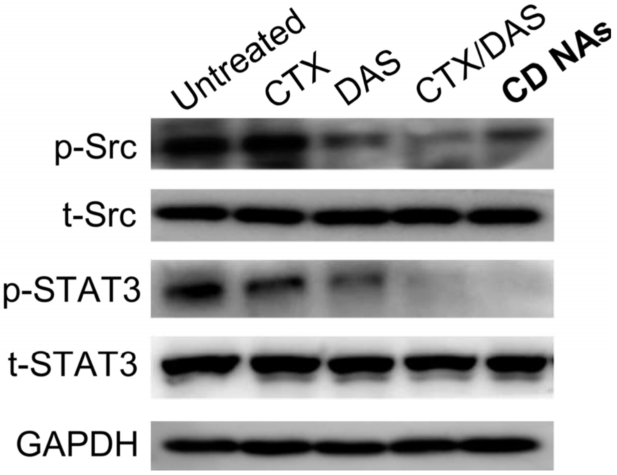

Figure S10. The downregulation of p-STAT3 expression by blocking the SRC signaling pathway in NCI-H1975 cells after treatment with **CD NAs** was monitored. After incubation with 1 nM CTX, 40 nM DAS, CTX+DAS (1 nM/40 nM) or **CD NAs** at identical concentrations, the cells were subjected to western blotting.

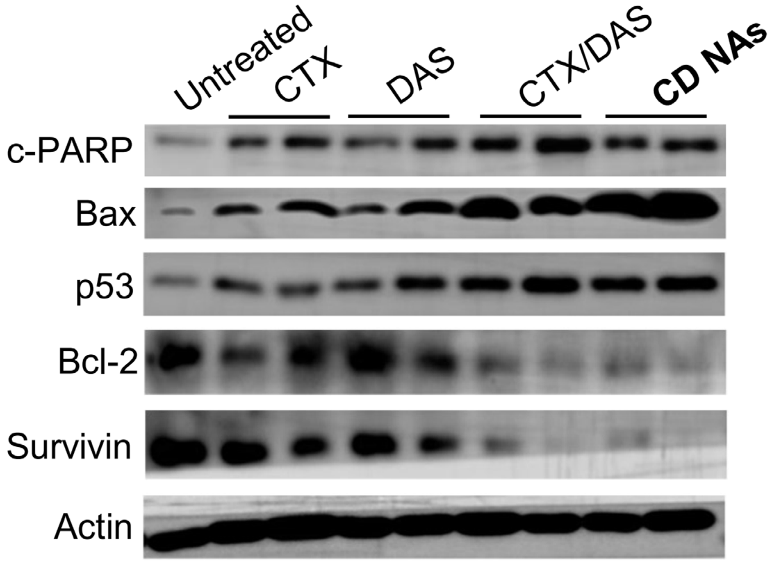

Figure S11. Proteins related to apoptosis were examined by western blotting in NCI-H1975 cells.

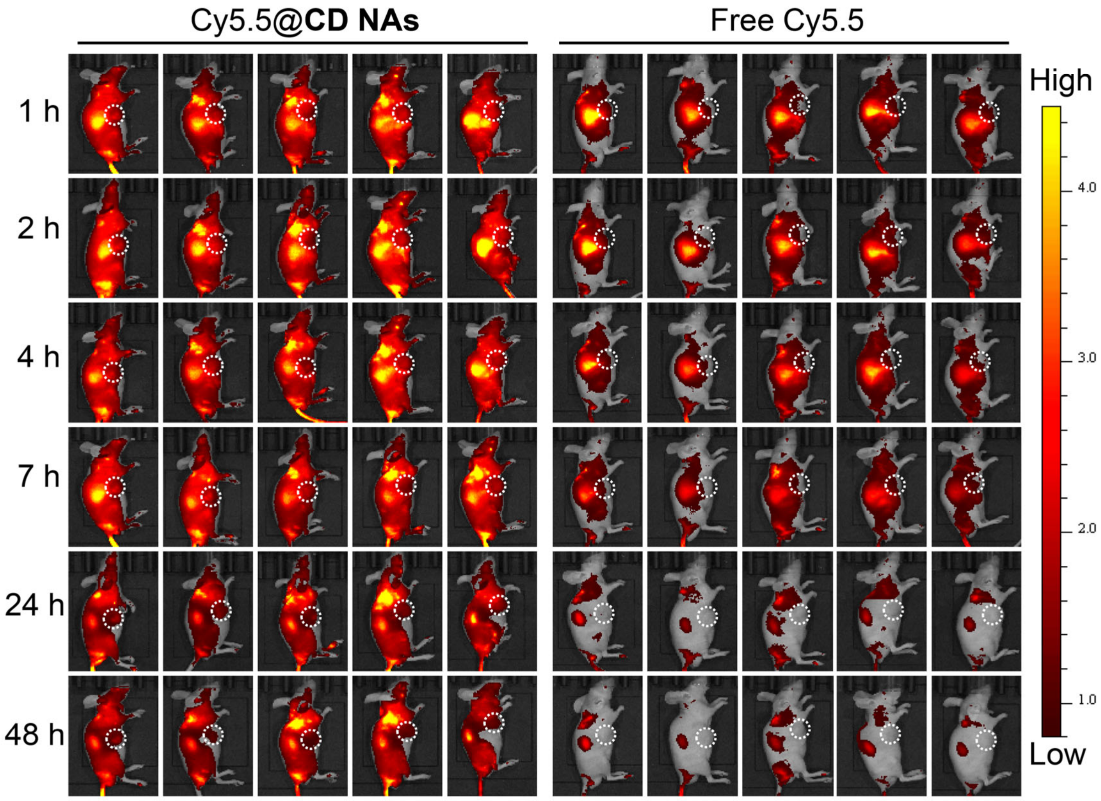

Figure S12. Real-time NIR fluorescence imaging of the nanoassemblies in NCI-H1975 tumor-bearing mice. Mice (n = 5 in each group) were intravenously injected with Cy5.5-loaded **CD NAs** (Cy5.5@**CD NAs**; 20 μg Cy5.5 per mouse) and measured by IVIS imaging at predetermined time points. Free Cy5.5 was used as control. The white dashed circles indicate the tumor regions.

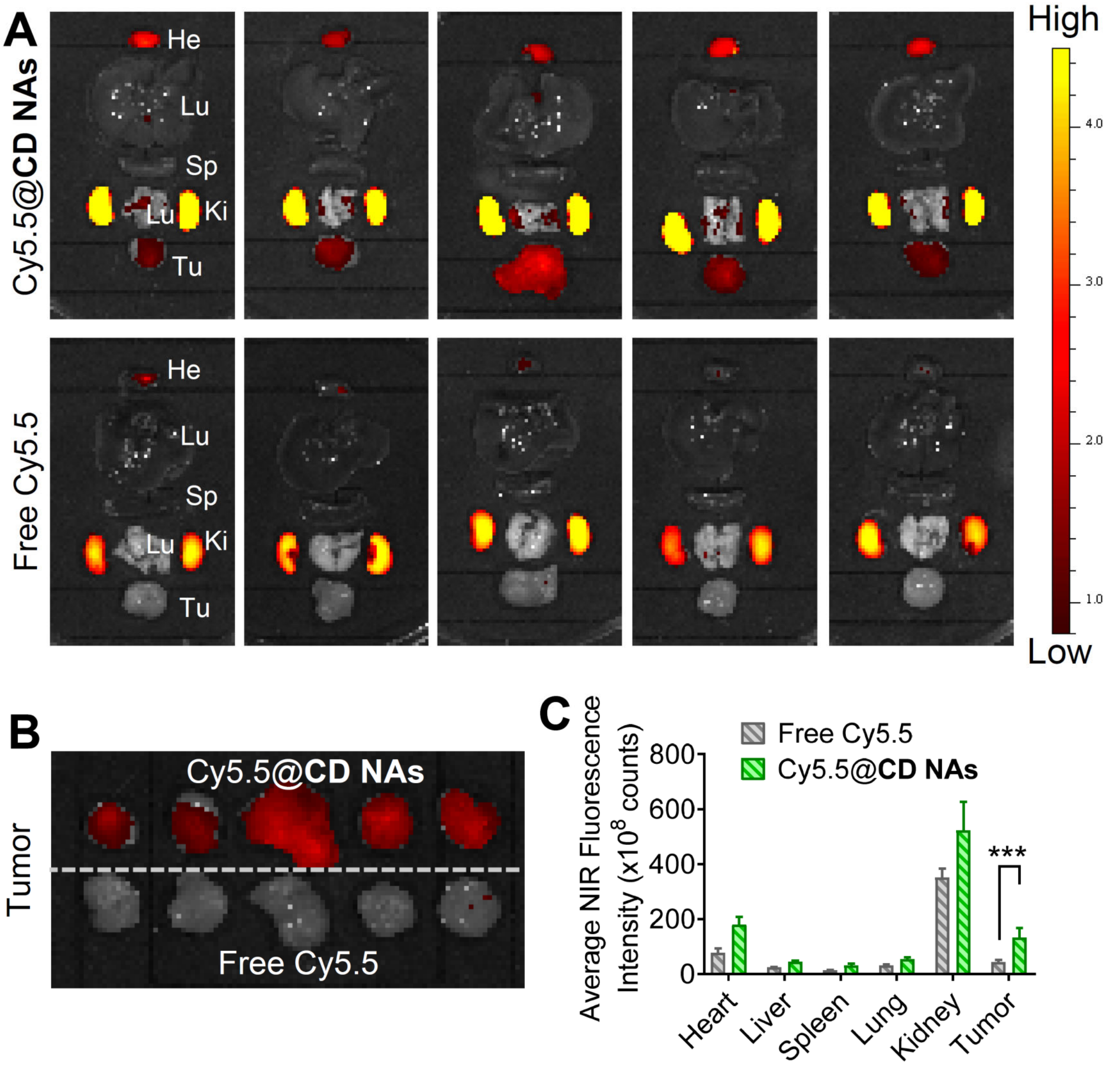

Figure S13. (A and B) *Ex vivo* fluorescence imaging of major organs and tumors. Mice were sacrificed at 48 h post-injection for analysis. He, heart; Li, liver; Sp, spleen; Lu, lung; Ki, kidneys; Tu, tumor. (C) Region-of-interest intensities of fluorescence signals among tumor and organs. The data are presented as the means ± SD (n = 5). ***$p < 0.001$.

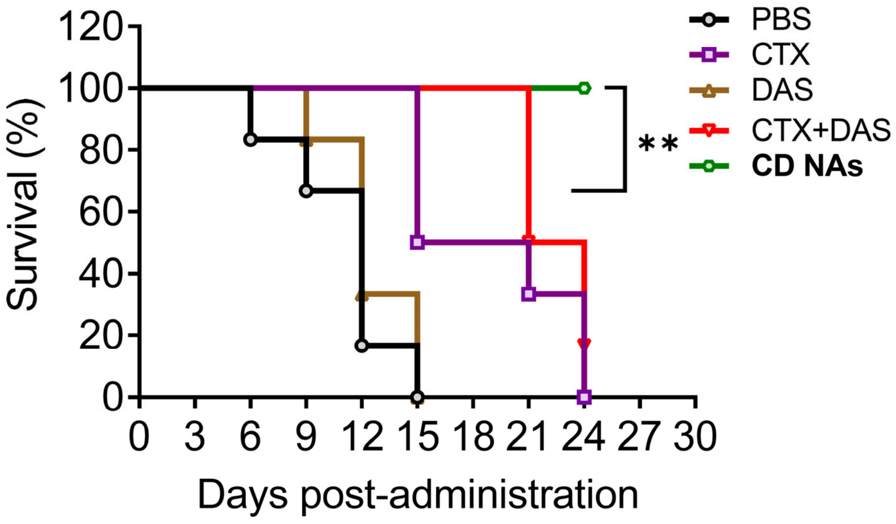

Figure S14. Survival percentage (Kaplan-Meier curves) of the mice bearing NCI-H1975 tumors after various treatments. The mice were defined to be dead when the tumor volume exceeded 500 mm^3. **p < 0.01.

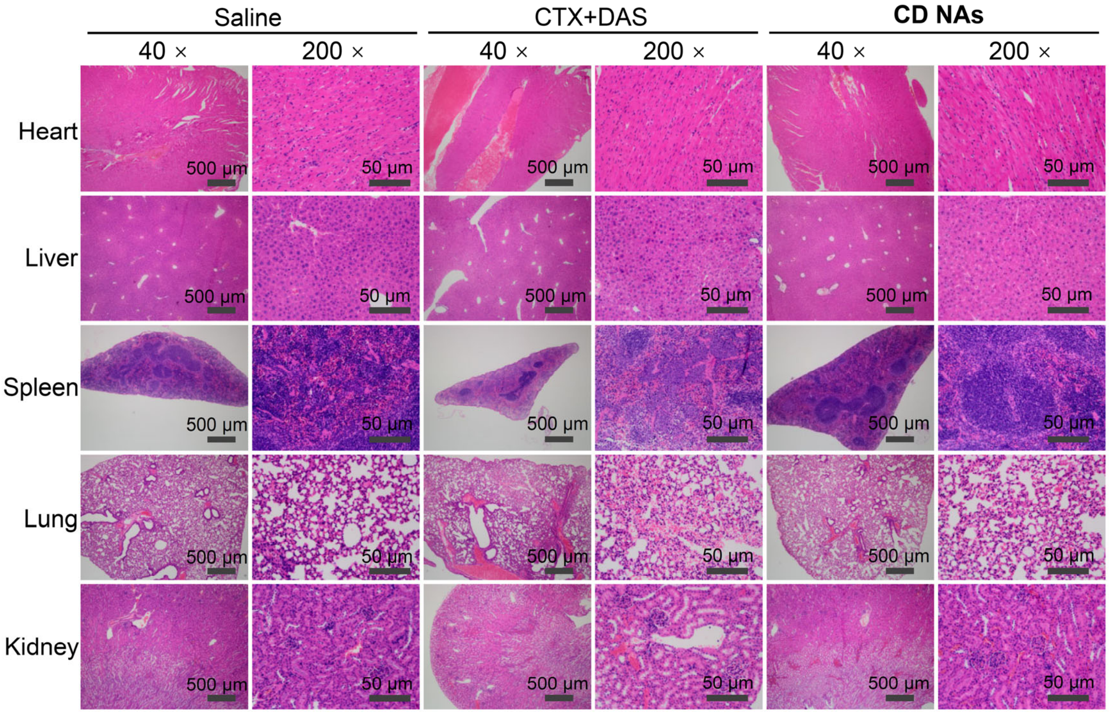

Figure S15. Histological analysis of major organs (heart, liver, spleen, lung, kidney) excised from healthy Balb/c mice on day 15 post-administration. Mice were administered free drug combination CTX+DAS (CTX: 6 mg/kg, i.v.; DAS: 30 mg/kg, *via* gavage) or **CD NAs** (i.v., 6 mg/kg CTX and 30 mg/kg DAS) for total five doses every three days. Saline was used as control. The image magnifications are 40 or 200.

Table S1. Characterizations of **CD NAs** at the varying weight ratios of CTX to DAS.

	Z-average [*d*, nm]	Zeta potential [mV]	PDI [b]	EE% [c]		DL%[d]
				CTX	DAS	
**CD NAs** (10:1)[a]	440.3±11.6	21.9±0.2	0.293±0.016	99.22	99.75	
**CD NAs** (5:1)	253.3±5.6	22.9±0.3	0.225±0.036	99.16	99.88	
**CD NAs** (1:1)	173.6±2.3	24.0±0.3	0.162±0.033	99.62	99.63	
**CD NAs** (1:5)	164.0±1.9	26.6±0.5	0.063±0.029	99.75	99.88	
**CD NAs** (1:10)	152.8±1.2	27.2±0.4	0.060±0.007	99.77	100.00	100%
**CD NAs** (1:20)	152.0±1.5	27.5±0.4	0.116±0.018	99.78	99.48	
**CD NAs** (1:30)	151.3±3.0	28.0±1.7	0.052±0.020	99.79	100.00	
**CD NAs** (1:40)	150.9±1.0	27.6±1.0	0.023±0.008	99.79	99.50	
**CD NAs** (1:50)	150.9±1.0	29.0±0.7	0.042±0.026	99.78	99.48	
DAS NAs	168.2±0.8	29.7±0.3	0.035±0.018	--	100.25	

[a] All measurements were performed in triplicate;

[b] PDI, polydisperse index;

[c] EE (%), encapsulation efficiency (%);

[d] DL (%), drug loading (%)

Table S2. IC_{50} values tested in NCI-H1975 and NCI-H1299 cells. The cells were treated with free CTX, free DAS, the mixture of CTX/DAS or **CD NAs** for 48 h.

IC_{50} (nM)	NCI-H1975	NCI-H1299
CTX	4.6	13.1
DAS	194.8	392.3
CTX/DAS (1:40)	1.1/42.2	--
**CD NAs** (1:40)	0.7/27.9	--
CTX/DAS (1:30)	--	3.5/103.9
**CD NAs** (1:30)	--	2.6/76.6

Table S3. Combination index (CI) on 50% inhibition of DAS and CTX on NCI-H1975 cells. CI values were calculated by Calcusyn 2.1 based on the results of CCK-8 assay. Cells were treated with drugs for 48 h.

Molar ratios of CTX to DAS	CI (50% inhibition)	
	CTX/DAS	**CD NAs**
1:40	0.393	0.231
1:30	0.505	0.358
1:20	0.481	0.358
1:10	0.535	0.406
1:5	0.532	0.367
1:1	0.557	0.293

Table S4. Hematology measurements for saline, free CTX, free DAS, free CTX+DAS and **CD NAs** treated mice. Samples were collected at baseline, days 4, days 9 and days 15. Three doses were administrated every three days. Values are expressed as means ± SD (n=4).

	Baseline	Days 4				Days 9				Days 15			
	Saline	CTX	DAS	CTX+DAS	**CD NAs**	CTX	DAS	CTX+DAS	**CD NAs**	CTX	DAS	CTX+DAS	**CD NAs**
WBC (10^9/L)	6.75 ± 0.31	2.85 ± 0.37	4.17 ± 0.49	1.75 ± 0.28	3.57 ± 0.42	1.90 ± 0.49	3.62 ± 0.94	1.45 ± 0.36	3.12 ± 0.15	9.30 ± 0.95	6.60 ± 0.59	8.02 ± 0.92	6.97 ± 0.28
NE (10^9/L)	0.52 ± 0.05	0.10 ± 0.00	0.40 ± 0.14	0.07 ± 0.05	0.17 ± 0.05	0.05 ± 0.05	0.80 ± 0.28	0.17 ± 0.05	0.17 ± 0.09	1.15 ± 0.05	1.35 ± 0.73	2.30 ± 0.95	0.85 ± 0.20
NE%	9.52 ± 2.55	2.90 ± 0.73	9.37 ± 4.60	3.00 ± 1.03	6.25 ± 1.27	2.42 ± 1.42	24.2 ± 2.3	11.0 ± 3.8	7.07 ± 2.2	13.0 ± 1.3	15.4 ± 4.8	27.6 ± 8.4	13.0 ± 1.2
LY (10^9/L)	6.02 ± 0.22	2.75 ± 0.37	3.77 ± 0.61	1.65 ± 0.28	3.40 ± 0.42	1.55 ± 0.17	2.55 ± 0.30	1.10 ± 0.27	2.65 ± 0.3	8.10 ± 0.90	5.70 ± 0.64	5.47 ± 0.26	6.25 ± 0.25
LY%	90.1 ± 2.6	95.1 ± 1.4	90.4 ± 4.5	92.4 ± 4.2	91.7 ± 1.9	94.8 ± 2.4	76.0 ± 4.6	74.2 ± 3.3	89.87 ± 2.8	86.8 ± 1.2	81.0 ± 9.3	66.4 ± 9.4	86.6 ± 1.1
RBC (10^{12}/L)	8.7 ± 0.3	8.65 ± 0.44	7.96 ± 0.46	7.30 ± 0.63	7.96 ± 0.26	8.55 ± 0.38	8.24 ± 0.05	6.92 ± 0.49	8.15 ± 0.5	8.92 ± 0.58	9.35 ± 0.40	8.11 ± 0.30	8.41 ± 0.25
HGB (g/L)	174 ± 6	171 ± 11	163 ± 4	165 ± 4	164 ± 8	169 ± 7	166 ± 4	141 ± 6	162 ± 9.05	166 ± 5	177 ± 6	142 ± 3	150 ± 1
HCT	45.0 ± 1.6	44.4 ± 2.0	41.1 ± 1.9	37.0 ± 2.9	40.0 ± 2.4	42.5 ± 1.5	41.5 ± 0.5	34.3 ± 2.4	39.8 ± 2.97	44.6 ± 1.8	46.7 ± 1.9	39.7 ± 1.1	41.8 ± 0.3
MCV (fL)	51.3 ± 1.2	51.3 ± 0.9	51.7 ± 0.8	50.1 ± 0.8	51.0 ± 0.7	49.7 ± 0.9	50.3 ± 0.6	49.4 ± 0.6	50.17 ± 0.53	50.0 ± 1.7	50.0 ± 0.7	49.0 ± 0.7	52.1 ± 0.7
MCH (pg)	19.8 ± 0.4	19.7 ± 0.6	20.5 ± 0.7	21.2 ± 0.5	20.4 ± 0.6	19.7 ± 0.6	20.1 ± 0.6	20.5 ± 0.4	20.22 ± 0.83	18.6 ± 0.7	19.0 ± 0.3	18.2 ± 0.3	18.8 ± 0.2
MCHC (g/L)	380 ± 16	385 ± 10	396 ± 10	423 ± 8	406 ± 14	396 ± 6	399 ± 8	416 ± 7	393 ± 3.36	372 ± 3	378 ± 6	359 ± 6	362 ± 2
RDW (%)	13.2 ± 0.2	12.8 ± 0.2	13.2 ± 0.2	12.9 ± 0.4	13.6 ± 0.2	13.0 ± 0.3	13.25 ± 0.34	13.7 ± 0.5	13.45 ± 0.31	14.0 ± 0.1	13.5 ± 0.4	14.9 ± 0.2	14.5 ± 0.1
PLT (10^9/L)	723 ± 55	887 ± 115	733 ± 100	697 ± 103	720 ± 67	794 ± 136	622 ± 104	739 ± 53	701.25 ± 42.55	528 ± 79	593 ± 124	358 ± 18	631 ± 169
PCT	0.47 ± 0.03	0.68 ± 0.09	0.47 ± 0.06	0.53 ± 0.09	0.49 ± 0.04	0.54 ± 0.13	0.40 ± 0.06	0.51 ± 0.02	0.47 ± 0.05	0.35 ± 0.05	0.37 ± 0.07	0.23 ± 0.01	0.42 ± 0.11
MPV (fL)	6.50 ± 0.24	7.00 ± 0.25	6.52 ± 0.20	7.15 ± 0.31	6.72 ± 0.09	6.77 ± 0.50	6.50 ± 0.08	6.92 ± 0.25	6.77 ± 0.18	6.77 ± 0.17	6.27 ± 0.15	6.85 ± 0.44	6.65 ± 0.12
PDW (fL)	11.5 ± 0.4	12.5 ± 0.3	11.7 ± 0.6	12.4 ± 0.5	11.8 ± 0.4	11.9 ± 1.3	11.7 ± 0.1	12.0 ± 0.3	11.55 ± 0.09	11.6 ± 0.6	11.6 ± 0.47	12.0 ± 0.6	11.6 ± 0.4

References:

[1] Tian YQ, Wu WC, Chen CY, Strovas T, Li YZ, Jin YG, et al. 2,1,3-Benzothiadiazole (BTD)-moiety-containing red emitter conjugated amphiphilic poly(ethylene glycol)-block-poly(epsilon-caprolactone) copolymers for bioimaging. J Mater Chem. 2010;20:1728-36.

[2] Wang JM, Cieplak P, Kollman PA. How well does a restrained electrostatic potential (RESP) model perform in calculating conformational energies of organic and biological molecules? J Comput Chem. 2000;21:1049-74.

[3] Martinez L, Andrade R, Birgin EG, Martinez JM. PACKMOL: A package for building initial configurations for molecular dynamics simulations. J Comput Chem. 2009;30:2157-64.

[4] Case DA, Cheatham TE, Darden T, Gohlke H, Luo R, Merz KM, et al. The Amber biomolecular simulation programs. J Comput Chem. 2005;26:1668-88.

[5] Xie K, Song SS, Zhou LQ, Wan JQ, Qiao YT, Wang M, et al. Revival of a potent therapeutic maytansinoid agent using a strategy that combines covalent drug conjugation with sequential nanoparticle assembly. Int J Pharmaceut. 2019;556:159-71.
